# Artificial Tendons’ Responsiveness to Mechanical Stress and Biological Performance Following Cork Extract Functionalization

**DOI:** 10.1021/acsabm.5c00449

**Published:** 2025-08-27

**Authors:** Bruna A. S. Oliveira, Marta O. Teixeira, Sónia P. Gonçalves, Artur Ribeiro, Carla Silva, Helena P. Felgueiras

**Affiliations:** † Centre for Textile Science and Technology (2C2T), 56059 University of Minho, Campus of Azurém, 4800-058 Guimarães, Portugal; ‡ Centre of Biological Engineering (CEB), 56059 University of Minho, Campus of Gualtar, 4710-057 Braga, Portugal; § LABBELS−Associate Laboratory, 4710-057 Braga, Guimarães, Portugal

**Keywords:** artificial tendons for hands, biodegradable and nonbiodegradable polymers, core−shell braiding, cork extract, multifunctional activity

## Abstract

Hand tendon ruptures have increased due to mechanical stress, degeneration, trauma, and repetitive motion. This study proposes an artificial tendon engineered from polymeric braids made of lyocell, biodegradable polyester, and polyethylene terephthalate, functionalized with natural cork extract. Cork extract 2, obtained by cork powder using hydroethanolic solvent in a planetary mixture, exhibited a phenolic content of 508.35 ± 82.26 mg GAE/g, flavonoid content of 607.32 ± 63.96 mg EQ/g, strong antioxidant activity (EC_50_ = 0.21 ± 0.02 mg/mL), and antimicrobial efficacy, with minimum bactericidal concentrations of 0.16 mg/mL against *Staphylococcus aureus* and 0.64 mg/mL against *Pseudomonas aeruginosa*. Single-polymer braids were processed with a core–shell structure, in which the core was formed of loose strands. The mechanical characterization of the braids showed the three polymers to exhibit an elongation at break of 8.72 ± 2.05–18.72 ± 6.58% and tensile strength of 44.22 ± 12.02–53.15 ± 12.62 MPa, all within desirable ranges for hand tendon repair/substitution. Braids were functionalized via physical adsorption of cork extract 2 (1.60 mg/mL), achieving loading values between 0.33 ± 0.16 and 0.55 ± 0.14 mg/mL. The presence of extract in the braids was confirmed via infrared spectroscopy and through thermal characterization, thermogravimetry, and differential scanning calorimetry. Functionalized braids exhibited controlled release of the cork extract 2, ranging from 1.57 ± 4.14 to 13.90 ± 2.02% within 24 h, ensuring sustained bioactivity, demonstrating as well strong antioxidant activity (87.42 ± 1.18 reduced 2,2-diphenyl-1-picrylhydrazyl), antimicrobial activity (achieving up to 99% reduction in *S. aureus* and 80% reduction in *P. aeruginosa*), and low cytotoxicity (>70% metabolic activity); with the functionalized lyocell presenting the best overall performance. In general, the proposed strategy demonstrated promise for hand tendon repair, offering a potential innovative solution to improve patients’ quality of life.

## Graphical abstract

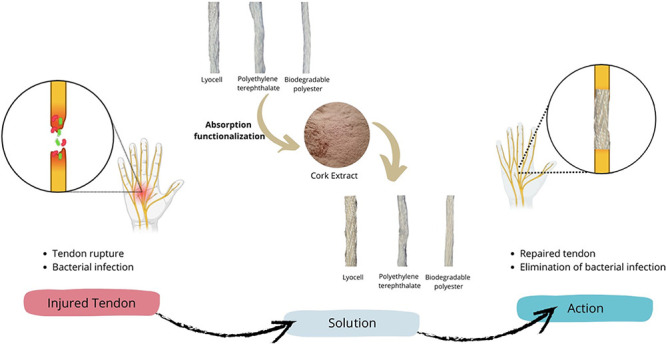

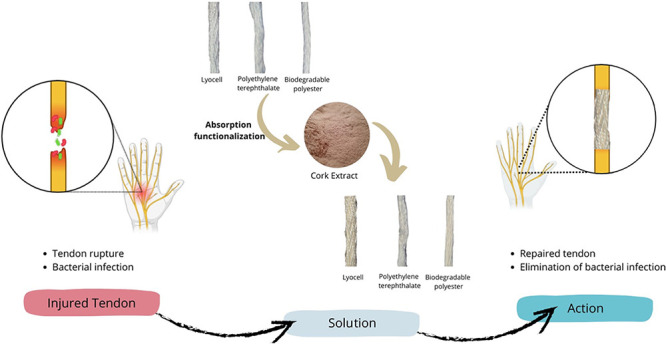

## 1 Introduction

Tendons consist of dense, fibrous connective tissue responsible for transmitting mechanical forces between muscles and bones, enabling joint movement. Tendon structure is highly organized and hierarchical, consisting of collagen molecules, fibrils, fibers, and fascicles, all aligned parallel to a geometric axis.^1^ The mechanical properties of human tendons, including those of the hand, vary depending on the specific type of tendon, age and sex of the individual. Indeed, tensile strengths at break can be found between 24 and 100 MPa, the Young’s modulus between 1 and 2 GPa, the toughness between 1000 and 4500 J/kg, and deformation between 4 and 10%.^2,3^

There has been a significant rise in the incidence of tendon ruptures in humans, with approximately 33 million musculoskeletal injuries being reported each year in the USA and nearly half of those involving tendons and ligament. Tendons are structures adapted to withstand high tension; yet, when exposed to repetitive or intense loads, they can sustain microinjuries which, without adequate recovery, may progress to structural and functional damage. Intrinsic factors, such as body weight, nutrition and age, also play a significant role. Tendon injuries can be categorized as chronic, known as tendinopathies, or acute, like ruptures. In chronic injuries, the main focus is on reducing pain, often using anti-inflammatory agents, whether administered locally or systemically. On the other hand, in acute injuries, the priority is to repair damaged tendons, usually through specialized surgical procedures.^4,5^ Grafts are such an approach and can be subdivided into three main categories: (1) autologous grafts, which involve the use of the patient’s own cellular tissue; (2) allografts, obtained from tissue donors; and (3) synthetic grafts which, despite having faced high rates of failure and synovial inflammation in the past, have sparked new interest in regenerative medicine.^6^

Hands are frequently subjected to injuries due to excessive use in daily life, professional tasks, and sports activities. Hand injuries account for approximately 14–30% of emergency interventions, with tendon damage representing the second most frequent cause (≈29%). Hand tendons are divided into two groups, the extensors and the flexors. The extensor tendons extend from the forearm, across the back of the hand, and reach the fingers, allowing extension and alignment of the fingers. On the other hand, the flexor tendons start from the forearm, pass through the wrist and reach the palm of the hand, allowing the fingers to be flexed. The characteristics of tendons vary depending on the specific type of tendon, gender, and age of the patient in question. In the study carried out by Ito et al., the tendon of the palmaris longus muscle was examined in 72 forearms from 36 cadavers. For males, the average length and thickness were 124.6 ± 17.0 and 4.5 ± 0.7 mm, respectively, and for females 108.3 ± 16.4 and 4.0 ± 0.7 mm, respectively.^7^ Which corroborates that there is a difference in the characteristics of having between genres.

Braids have been gaining relevance in the rehabilitation of soft musculoskeletal tissue, including tendons and ligaments, as they guarantee mechanical properties, namely stress–strain behavior, close to the original. In fact, surgical treatments have adopted braiding-related approaches, because of the resistance and flexibility of the structures obtained.^8^ Through braiding, threads intertwine diagonally in relation to the main axis of the structure, forming a basic pattern, known as a diamond pattern, in which the threads alternate above and below, generating a cylindrical structure.^9^ Braids can also be produced with a core, for example with another braid or loose threads as filling, and the threads intertwining entrap that material, or they can be hollow, without any filling. The speed of bradding system plays a crucial role, determining the angle of the braid and influencing its properties. At high speed, the braid has a smaller angle and, consequently, becomes more open.^9^ The opposite occurs at lower speeds, where the resulting braid is more compact and presents an obtuse angle.^9^

The search for ecologically sustainable alternatives in the biomedical area is a challenge for the current ages.^10,11^ In this context, lyocell and biodegradable polyester (BP) emerge as promising options for the development of artificial tendons. Lyocell is a fiber made up of regenerated cellulose, a natural polymer present in the cells of all plants, extracted from the pulp of tree wood, mainly eucalyptus. Lyocell results from spinning a solution of cellulose in *N*-methylmorpholine-N-oxide (NMMO), a nontoxic solvent, through a wet-jet dry spinning apparatus.^12,13^ This polymer has excellent properties, such as biodegradability, absorption capacity, high tensile strength, flexibility, high purity and excellent tenacity (amount of energy it absorbs before breaking). However, the lack of intrinsic antibacterial properties limits its application in medical contexts.^12,13^ On its turn, CRAFTEVO ReTE is a type of biodegradable polyester made from terephthalic acid and ethylene glycol, the two primary components of polyester, sourced from biobased materials (1:ISCC:Plus, mass balance), thereby reducing reliance on petroleum. This material is made to maintain the same durability and heat resistance as common polyester, with a melting point of approximately 235 °C (polyester melting point is at 260 °C). However, like lyocell, possesses limited antibacterial properties, which can be a concern for applications where microbial resistance is essential. Despite not being ecologically sustainable, polyethylene terephthalate (PET) has garnered attention for its biomedical potential. This thermoplastic polymer, derived from petroleum, is characterized by a complex structure that provides notable resistance to degradation, making it suitable for applications in long-term use medical devices. Essentially composed of terephthalic acid and ethylene glycol, PET stands out for its durability, lightness, high transparency, for its nonabsorbable nature, and high tensile and impact resistance. It has demonstrated good biocompatibility in medical implants, particularly with an open weave structure that allows for tissue ingrowth, as seen in designs like the artificial Ligament Advanced Reinforcement System (LARS). However, its hydrophobicity and limited in vivo biocompatibility tend to hinder PET’s broad biomedical application.^14^ In this sense, modifying the surface of these polymers for improving their biological performance becomes essential for achieving the desired features for hand tendon repair.

Cork extract stands out for its antioxidant and antimicrobial potential. It can be obtained from the bark of the cork oak tree, *Quercus suber*, belonging to the *Fagaceae* family and is characterized by its renewable, nontoxic and recyclable nature. Cork contains bioactive compounds beneficial to health, namely suberin, lignin and cellulose, and small amounts of extractives, terpenes, fatty acids, phenolic acids, tannins, long-chain aliphatic compounds, saccharides, etc.^15^

This study focuses on the development of an innovative fibrous system functionalized with cork extract to enhance tendon repair strategies. By integrating biodegradable materials such as lyocell and BP with the antimicrobial and antioxidant properties of cork, this research aimed at addressing the limitations of current grafting options while promoting ecologically sustainable alternatives. Comparisons were drawn against the nonbiodegradable PET. The unique combination of these materials is expected to improve both the mechanical and biological properties of tendon repair constructs. Comprehensive evaluations of mechanical performance, structural integrity, and biological compatibility were conducted to assess the efficacy of the developed system. Ultimately, this approach sought to provide a robust solution for tendon injuries, hence, leading to improved recovery outcomes for patients and contributing to advancements in regenerative medicine.

## 2 Materials and Methods

### 2.1 Reagents, Bacterial Strains, and Cell Line

Folin-Ciocalteu reagent, quercetin (98%), gallic acid, 2,2-diphenyl-1-picrylhydrazyl (DPPH), Trolox (6-hydroxy-2,5,7,8-tetramethylchroman-2-carboxylic acid; 97.0%), sodium nitrite (NaNO_2_; 97.0%), Dulbecco’s modified Eagle medium (DMEM), medium 199 and hygromycin were purchased from Sigma-Aldrich, Germany. Sodium carbonate (Na_2_CO_3_) was purchased from Riedel-de Haën, Germany, aluminum chloride (AlCl_3_) from Acros Organics, USA, and sodium hydroxide (NaOH) from Labkem, Spain. Diiodoethane (99%) were obtained from Alfa Aesar, USA. Dimethyl sulfoxide (DMSO) and ethanol (99.8%) were purchased from Fisher Scientific, United Kingdom. 2,4,6-Tri­(2-pyridyl)-1,3,5-triazine (TPTZ; 98%) was supplied by Thermoscientifc, Germany. Acetic acid and ethylene glycol were purchased from Panreac, from Spain. Crystal violet was purchased from Certistain, USA. Dopamine and methanol were obtained from Merk KGaA and tris hydrochloride from Roche, both from Germany. *Staphylococcus aureus* (ATCC 6538), *Pseudomonas aeruginosa* (ATCC 27853) and BJ-5ta cell line were obtained from the American Type Culture Collection (ATCC, USA). Mueller Hinton broth (Mueller Hinton Broth, MHB) was purchased from Pronadisa (Condalab, Spain), while trypticase soy broth (TSB) and soy agar tipticase (trypticase soy agar, TSA) were from HiMedia Laboratories (Germany). These culture media were used for bacterial growth. Nutrient broth (NB) and nutrient agar (NA) were purchased from VWR Chemicals (USA) and were used as culture media for the growth of *Pseudomonas aeruginosa* bacterium. The phosphate-buffered saline (PBS) used at 0.01 M was prepared at pH 7.4 from the combination of sodium chloride (NaCl; 8.00 g/L), potassium chloride (KCl; 0.20 g/L), sodium phosphate dibasic (Na_2_HPO_4_; 1.44 g/L) and monopotassium phosphate (KH_2_PO_4_; 0.24 g/L), all obtained from Sigma-Aldrich. Fetal bovine serum (FBS), trypsin and penicillin/streptomycin solution were purchased from Grisp (Portugal). Reagents were all used as commercially available, without additional chemical modifications.

### 2.2 Extraction of Bioactive Compounds from Cork

A bioactive extract was obtained from cork powder (Cork Supply, Portugal) using a hydroethanolic solution, in a 50:50 (v/v) ratio, and a planetary mixer ARE 250 (THINKY, UK). Two extraction methods were applied, and for each method, the cycle was repeated 3 times and 4 successive extractions were made (Table 1). In extraction method 1, the solvent was renewed at the end of each extraction, while in extraction method 2, the raw material was renewed at the end of each extraction. From this moment on, nomenclatures were assigned to the extract obtained from each method: cork extract from method 1 (C1) and cork extract from method 2 (C2).

**Table 1:** Extraction Conditions Used in the Planetary Mixer

natural source	natural source: solvent ratio (g: mL)	methods (steps)
cork	5:100	(1) 1000 rpm–5 min
		(2) 2200 rpm–1 min
		(3) 2000 rpm–5 min
		(4) 2200 rpm–1 min
		(5) 2000 rpm–5 min
		(6) 2200 rpm–1 min
		(7) 2000 rpm–5 min
		(8) 2200 rpm–1 min
		(9) 1000 rpm–5 min
		total time for each cycle: 29 min
		total time for one extraction: 116 min
		total extraction time: 464 min

After each method, the extract was filtrated and transferred to a rotary evaporator (Heidolph, German) working at 45 °C and 70 rpm. Finally, the samples were freeze-dryer (CoolSafe, Denmark) and the extractions yields (%) calculated according to eq 1:

$$\mathrm{Extraction}\;\mathrm{yield}=\frac{W_{2}}{W_{1}}\times 100$$ 1

where *W*_1_ is the weight (g) of the initial cork powder sample and *W*_2_ is the final weight (g) of the freeze-dried product after each extraction method.

### 2.3 Biological Characterization of the Cork Extracts

#### 2.3.1 Determination of Total Phenolic Content

The total content of phenolic compounds (TPC) was determined using an adaptation of the Folin-Ciocalteu method. Two μL of extract (1 mg/mL) dissolved in DMSO were combined with 10 μL of Folin’s reagent and 120 μL of distilled water (dH_2_O), in the dark. The mixture was homogenized and further combined with 40 μL of Na_2_CO_3_ at 15% w/v and 28 μL of dH_2_O. After further homogenization, the microplate was kept in the dark for 2 h at room temperature (RT). Absorbance was then measured at 750 nm (SYNERGY|H1, BioTek, US). The amount of phenolics was calculated using a standard gallic acid calibration curve and using DMSO as a negative control. Results were expressed in mg of gallic acid equivalents (GAE)/g of sample dry weight (mg GAE/g extract). TPC was determined using eq 2:

$$\mathrm{TPC}=C_{\mathrm{GA}}\times \frac{V}{m}$$ 2

where *C*_GA_ corresponds to the concentration of gallic acid obtained from the calibration curve (mg/mL), *V* represents the extract volume (mL), and *m* corresponds to the mass of the extract (g).

#### 2.3.2 Determination of Total Flavonoid Content

Total flavonoid content (TFC) was determined based on the aluminum chloride colorimetric method, with modifications. Briefly, 17.5 μL of extract (1 mg/mL) dissolved in DMSO were mixed with 140 μL of dH_2_O and 5.25 μL of NaNO_2_ at 10% w/v, protected from light. After 5 min, 5.25 μL of AlCl_3_ at 20% w/v, 70 μL of NaOH at 1 M and 112 μL of dH_2_O were added. Absorbances were measured at 510 nm (SYNERGY|H1, BioTek, US) at RT using DMSO as negative control and quercetin as positive control (calibration curve). Results were expressed in mg of quercetin equivalents (QE)/g of sample dry weight (mg QE/g extract). TFC was determined using eq 3:

$$\mathrm{TFC}=C_{Q}\times \frac{V}{m}$$ 3

where *C*_Q_ corresponds to the concentration of quercetin obtained from the calibration curve (mg/mL), *V* represents the extract volume (mL), and *m* corresponds to the mass of the extract (g).

#### 2.3.3 Determination of Antioxidant Activity via DPPH Free Radical Scavenging Assay

The extracts antioxidant activity was evaluated using an adaptation of the DPPH assay. DPPH was prepared at 200 μM in 99.8% v/v ethanol and stored at 4 °C, protected from light. The extract was dissolved in a hydroethanolic solution at concentrations of 5.0, 10.0, 50.0, 100.0, 150.0, 200.0, and 500.0 μg/mL. Then, 10 μL of extract were combined with 140 μL of DPPH solution. The absorbance was monitored continuously every 5 min for 1 h, at RT, and 515 nm (SYNERGY|H1, BioTek, US). Here, the hydroethanolic solution was used as a negative control, while Trolox was used as positive control and for the preparation of the DPPH reduction calibration curve.

The maximum effective concentration (EC_50_) was determined from the percentage of DPPH reduced, representing the concentration necessary to reduce DPPH discoloration by 50%. The antioxidant activity of the extracts was expressed in Trolox equivalents (TE) per 1 g of dry weight sample (mg TE/g extract), according to eq 4:

$$\mathrm{TE}=\frac{{\mathrm{EC}}_{50}\mathrm{Trolox}}{{\mathrm{EC}}_{50}\mathrm{Extract}}$$ 4

#### 2.3.4 Determination of Minimum Inhibitory and Minimum Bactericidal Concentrations

Preinoculum of *S. aureus* and *P. aeruginosa* were prepared for 18 h at 37 °C and 150 rpm. The extracts minimum inhibitory concentrations (MICs) were determined using the microdilution method based on the Clinical and Laboratory Standards Institute (CLSI) and the European Committee on Antimicrobial Susceptibility Testing (EUCAST).^16,17^ Stock solutions of each extract at an initial concentration of 20.48 mg/mL were prepared in dH_2_O. 50 μL of MHB and 100 μL of each extract solution were added to column 1 of a 96-well plate (in triplicate). Serial dilutions of 1/2 (v/v) in MHB were subsequently performed. Finally, 50 μL of bacterial suspensions, prepared at a concentration of 1 × 10^6^ colony forming units (CFUs)/mL, were added to each well. MHB (negative) and the extract-free bacterial suspension (positive) were used as control. Absorbances were measured with an EZ READ 2000 microplate reader (Biochrom, Cambridge, United Kingdom) at 600 nm, at time 0 h and after 24 h incubation at 37 °C and 120 rpm. MICs were determined by the difference in absorbance readings. On its turn, the minimum bactericidal concentrations (MBCs) were identified by culturing the bacterial suspensions at MIC and at concentrations before and after MIC. To this end, aliquots were collected and diluted in PBS in ratios of 1/10 (v/v; 10^–1^–10^–4^). The dilutions were then plated in agar (TSA for *S. aureus* and NA for *P. aeruginosa*) by the teardrop method. After 24 h at 37 °C, the observed colonies were counted.

### 2.4 Braids Production

The production of artificial tendons from lyocell 29.5 tex, with twist 14 turns per inch (*v*/″) (Risatel, Portugal), BP 14.7 tex and twist 5 *v*/″ (Grupo Valérius, RDD, Portugal), and 33 tex multifilament PET (Mundifios, Portugal) comprised two distinct stages: coiling and braiding.

Sixteen coils were initially filled with yarn at a speed of 50 rad/s (radian per second) using a parallel winding winder, model PR/810 (Trenz-Export, Spain). Subsequently, the yarn-filled coils were placed in a vertical braider, model 16/100 (Trenz-Export, Spain), and production was initiated. Braids were prepared with varying winding speeds, 3.0 and 4.0 rad/s, so a more open or closed braiding structure could be attained. Furthermore, braids were produced with 16 threads, either without a core (empty filling) or with a core composed of loose threads or smaller braids (4, 8, 16, or 32 threads). A total of 6 conditions were used for braids’ production using each material.

#### 2.4.1 Assessment of Mechanical Performance of the Engineered Braids

Tensile strength tests were conducted using a 10KS dynamometer (Hounsfield, UK), with HT400 pneumatic grippers (Tinius Olsen, UK). The standard adopted for tensile strength assessments was the EN 29073 3:1992–*Determination of tensile strength and elongation*. A 2.5 kN load cell was used and a pretension of 10 N was applied. For each braid sample, 15 measurements were carried out, maintaining a speed of 100 mm/min and a gauge length of 10 cm. The three braids that presented characteristics closer to those of human tendons (in terms of elongation, %, and breaking strength, MPa; the latter calculated taking into account the cross-sectional area of each braid), advanced to the following testing stages.

### 2.5 Pretreatment of Braids

In order to eliminate potential paraffin residues arising from the sizing/enzymatic treatment of the yarns, the prepared braids were washed three times in water at 500 rpm, at a temperature of 90 ± 5 °C for 30 min, and at a temperature of 60 ± 5 °C for another 30 min (procedure recommended by Risatel Company).

The effectiveness of the washing process was determined by attenuated total reflectance-Fourier transform infrared spectroscopy (ATR-FTIR) using the IRAffinity-1S, Shimadzu (Kyoto, Japan), coupled to an ATR accessory with diamond crystal (Specac, Japan). Evaluations were carried out under the following conditions: 200 scans per sample at a resolution of 2 cm^–1^, and measurement between 400 and 4000 cm^–1^, in three samples. Additionally, thermal degradation of the braids before and after washing was mapped using thermogravimetry (TGA). Measurements were carried out in a temperature range of 25–500 °C with an increasing range of 10 °C/min, under a 200 mL/min nitrogen atmosphere using the STA 7200 Hitachi (Fukuoka, Japan). The initial mass of each sample was 5 ± 2 mg. The results were plotted as mass loss (%) by temperature (°C).

The effect of the washing process and the presence of moisture on the braids’ mechanical performance was verified as described in Section 2.4.1. The list of samples/conditions tested was as follows: (1) unwashed and dried braids (results acquired in the preliminary tests described in Section 2.4.1); (2) unwashed and wet braids (immersion for 2 h in 0.01 M PBS at pH 7.4); (3) washed and dried braids; and (4) washed and wet braids.

### 2.6 Braids Functionalization with Cork Extract

Braids were functionalized with cork extract (selected based on data from Section 2.3) via physical adsorption. The extract calibration curve was prepared in dH_2_O at concentrations of 50.0, 32.0, 24.0, 16.0, 11.0, 10.0, 5.0, and 2.0 μg/mL, and the absorbance was measured using the ultraviolet–visible (UV–vis) spectrophotometer TCC-240 A (SCHIMADZU, Japan), scanning between 190 and 400 nm, with an interval of 1 nm. Functionalization was optimized with higher concentrations of extract (2 × MBC, 5 × MBC and 10 × MBC), to ensure that the amount effectively bound to the braids was equal to or greater than the MBC. The physical adsorption method involved immersing the samples in dH_2_O for 24 h at 150 rpm (prehydration), followed by another 24 h in extract solution (ratio 1:1 cm/mL; e.g. sample of 1 cm length immersed in 1 mL solution). The test was performed at RT. After 24 h of contact with the extract, the samples were washed 3 times with dH_2_O for 5 min at 150 rpm, to remove potential unbound extract molecules. Absorbances of all solutions (extract and washings) were read using the UV–vis spectrophotometer TCC-240 A (SCHIMADZU, Japan), between 190 and 400 nm. The functionalization capacity was determined indirectly based on the extract content remaining and the number of molecules released after three washes (weakly bound to the braids).

### 2.7 Braids Physical, Chemical, Thermal, and Mechanical Characterization

#### 2.7.1 Morphology

The braids morphology was captured by digital photography using a Samsung Galaxy S24 Ultra Smartphone at a distance of 9 cm from the lens and magnification of 4.2×, in the presence and absence of the extract. The thickness of the tendon-like structures was analyzed pre- and postfunctionalization of cork extract, both in dry and wet states, using a caliper (Mitutoyo, Japan).

#### 2.7.2 Chemical Groups’ Identification

The identification of the chemical groups’ characteristic of each of the engineered braids, pre- and postextract functionalization, was carried out by ATR-FTIR using the IRAffinity-1S, Shimadzu (Kyoto, Japan) as described in Section 2.5. The samples were left protected from the environment before carrying out the analysis, to reduce the influence of the water molecules present in the air.

#### 2.7.3 Thermal Response

The thermal profile of the braids before and after cork extract functionalization was analyzed by thermogravimetry (TGA), following the conditions described in Section 2.5, and differential scanning calorimetry (DSC). DSC data were collected using a DSC-600 from PerkinElmer (Columbus, USA) and samples of 5 ± 2 mg sealed in aluminum crucibles, subjected to progressive heating (0–400 °C) at a rate of 10 °C/min, under a dynamic nitrogen atmosphere at 20 mL/min (inert environment). Degradation temperatures (*T*_d_), melting temperatures (*T*_m_) and enthalpies (Δ*H*) were determined.

#### 2.7.4 Wettability and Surface Free Energy

Contact angle measurements were conducted using an OCA 200 goniometer, Data Physics (Filderstadt, Germany), connected to the OCA15 plus software (version 1.2), as described in the ASTM-D7334-08 standard. To that effect, 2 μL droplets of ultrapure water were deposited at a speed of 2.0 μL/s on the samples. Five measurements were carried out per sample, with the angles being recorded immediately after the droplet came into contact with the surface. In turn, the surface free energy (SFE) was calculated based on the angles obtained with ultrapure water (polar solvent), ethylene glycol (nonpolar solvent) and diiodomethane (nonpolar solvent) also using 2 μL drops (solvent information in Table 2). Once again 5 measurements were carried out on the surface of the samples at 22–23 °C and relative humidity of ≈50%, for each liquid and sample type. The Owens and Wendt method was used to calculate the SFE (eq 5):

$$\frac{\gamma (1+\cos\;\theta )}{2\sqrt{\gamma^{d}}}=\sqrt{\gamma {s^{p}}^{*}}\left(\frac{\sqrt{\gamma^{p}}}{\gamma^{d}}\right)+\sqrt{\gamma s^{d}}$$ 5

where γ is the total surface tension of the sample liquid with dispersive γ^d^ and polar γ^p^ components, and γ^s^ is the surface free energy of a solid with dispersive γ*s* ^d^ and polar γ*s* ^p^ components. θ is the contact angle between the liquid and solid surface of the sample (average of 5 measurements).

**Table 2:** Surface Tension (γ^t^) and Dispersive (γ^d^) and Polar (γ^p^) Components for Determining SFE, Using the Solvents: Ultrapure Water, Ethylene Glycol, and Diiodomethane^18^

liquid	γ^d^ (mN/m)	γ^p^ (mN/m)	γ^t^ (mN/m)
ultrapure water	21.8	51.0	72.8
ethylene glycol	29.0	19.0	48.0
diiodomethane	50.8	0.0	50.8

#### 2.7.5 Hydration and Water Retention Capacities

The hydration capacity (H) and the percentage of water retention (WR) by the braids were evaluated using samples measuring 1 cm in length were submerged in 1 mL of 0.01 M PBS for periods of 2, 4, 6, 24, and 48 h (when saturation was reached), at a constant incubation temperature of 37 °C. Braids were weighed before and after each immersion period, with excess PBS being removed using kimwipes (Kimtech). H was determined using eq 6:

$$H(\%)=\frac{m_{w}-m_{d}}{m_{w}}\times 100$$ 6

where *m*_w_ (mg) represents the mass of the wet braids after each incubation period and *m*_d_ (mg) represents the mass of the braids in the dry state, before immersion in PBS.

The percentage of WR was evaluated using eq 7:

$$\mathrm{WR}(\%)=\frac{m_{w}}{m_{d}}\times 100$$ 7

where *m*_w_ (mg) represents the mass of the wet braids after each incubation period and *m*_d_ (mg) represents the mass of the braids in the dry state, before immersion in PBS.

#### 2.7.6 Porosity

The porosity index (*P*) of the braids was determined by the amount of solvent absorbed after 1 h of immersion in 99% ethanol, under static conditions, using eq 8 (2 cm long samples):

$$P(\%)=\frac{W_{w}-W_{d}}{d_{\mathrm{ethanol}}V_{\mathrm{braid}}}\times 100$$ 8

where *W*_w_ is the weight of the wet braid and *W*_d_ is the weight of the dry braid, *d*_ethanol_ is the density of ethanol at RT (0.789 g/cm^3^) and *V*_braid_ is the volume of the wet braid. The *V*_braid_ was determined based on thickness measurements using a caliper and assuming the three-dimensional shape of a perfect cylinder for the calculations.

#### 2.7.7 Mechanical Performance

Tensile tests were conducted as described in Section 2.4.1, to determine the effect of the extract on the mechanical strength of the braids. Data were reported as elongation and tensile strength at break, representing the maximum deformation and load capacity before sample failure.

#### 2.7.8 Degradation

The degradation profile of the braids over a period of three months was analyzed under physiological-like conditions. Briefly, samples of 5 m in length (dimension required for conducting mechanical examinations; *n* = 15) were immersed in 1 L of 0.01 M PBS (pH 7.4) and incubated at 37 °C. Every 7 days, the PBS solution was replaced with a fresh one. Initially, the samples were washed (Section 2.5) and dried at RT to determine the initial dry mass (*m*_i_). After 1, 2, and 3 months of incubation, excess PBS was removed and the braids were washed 3 times, for 15 min each and agitation at 150 rpm for removing potentially adhered salts. The level of degradation induced by the physiological-mimicking environment was determined based on mass variations (eq 9):

$$\mathrm{weightloss}(\%)=\frac{m_{i}-m_{f}}{m_{i}}\times 100$$ 9

where *m*_i_ (g) is the mass of the sample on day 0 (after washing and drying) and *m*_f_ (g) is the mass of the dry sample after each incubation period.

Additionally, thermal examinations (Section 2.7.3) and mechanical evaluations (Section 2.4.1) were conducted after each incubation period.

### 2.8 Release Kinetics of Cork Extract from Functionalized Braids

The release of cork extract was mapped via UV–vis TCC-240 A spectroscopy (SHIMADZU, Japan), at a wavelength range between 190 and 400 nm. Braids of 1 cm in length, functionalized or extract-free, were incubated in 1 mL of 0.01 M PBS (pH 7.4) at 37 °C and 120 rpm, for 1, 2, 4, 6, 24, and 48 h. At each time point, 1 mL aliquots were collected, and 1 mL of fresh PBS were added. Absorbances were translated into mass using a cork calibration curve (Section S3 in Supporting Information) and taking into account the initial loading mass of the extract. Tests with extract-free samples were carried out to serve as a control, thus eliminating the influence of the polymers on the measurements (potential release of braid residues).

### 2.9 Antioxidant Activity

The braids antioxidant activity was evaluated following the procedure described in Section 2.3.3, with some modifications. To this end, samples were placed in contact with the DPPH solution (200 μM in 99.8% ethanol), ratio 1:1 (cm/mL), at 37 °C and 120 rpm, protected from light for 0, 1, 2, 4, 6, 24 h. At each time point, aliquots were collected and absorbances were measured at 515 nm (SYNERGY|H1, BioTek, US). DMSO was used as a negative control and Trolox as a positive control.

### 2.10 Antibacterial Activity: Time-Kill Kinetics Studies

For quantitative antibacterial evaluation, the “shake flask” method (ASTM-E2149) was used. Both functionalized and extract-free samples were sterilized in 70% ethanol and washed 3 times in sterile dH_2_O, each for 5 min at 120 rpm. Then, the samples were immersed in a bacterial suspension of *S. aureus* and *P. aeruginosa* (adjusted to a concentration of 1 × 10^5^ CFUs/mL and a ratio of 1:1 cm/mL) and incubated at 37 °C and 120 rpm, for 1, 2, 4, 6, and 24 h. At each interval, aliquots were collected and serial dilutions in PBS (10^–1^–10^–6^) were conducted. Finally, the solutions were plated on agar using the teardrop method and incubated at 37 °C for 24 h. The grown colonies were counted, and the results were expressed as percentage of bacterial reduction.

### 2.11 Cell Cytotoxicity Evaluations

Cytotoxicity tests were performed using the BJ-5ta cell line (normal human fibroblasts immortalized by overexpression of telomerase), which was cultured in a growth medium composed of 4 parts of DMEM containing l-glutamine, d-glucose, sodium bicarbonate. One part of medium 199, necessary for the growth of these cells, was also added. The final medium was supplemented with 5% fetal bovine serum (FBS), 1% (v/v) penicillin/streptomycin solution and 10 μg/mL hygromycin. Cell subcultures were initiated when confluence reached ≈80 to 90%. BJ-5ta cells were maintained in 75 cm^2^ tissue culture flasks in a 37 °C incubator in a humidified atmosphere of 5% CO_2_ in air. Cell culture medium was renewed twice a week. For subcultures and plating, adherent cells were detached with 0.05% trypsin solution, and fresh medium was added to neutralize the trypsin. The cell suspension was centrifuged for 5 min at 160 *g*. The supernatant was discarded, and fresh medium was added to obtain a new cell suspension, which was loaded into a Neubaeur chamber and the cell concentration estimated.

The cytotoxicity of the braids with and without cork extract functionalized was evaluated by direct contact, exposing BJ-5ta cells to the braids for 24 and 48 h. The braids were sterilized by UV for 1 h and in 100% ethanol for 20 min, followed by several washes in sterile 0.01 M PBS at pH 7.4. Braids were equilibrated with culture medium for 10 min before being added to the wells containing BJ-5ta cells. Cells were seeded at a density of 1 × 10^5^ cells/500 μL/well in a 24-well tissue culture plate the day before the assay. Cells were then exposed to the braids and incubated at 37 °C in a humidified atmosphere of 5% CO_2_ in air. Cells incubated with 30% DMSO were used as negative control, and cells incubated with culture medium without contact with braids were used as positive control. At the end of 24 and 48 h of contact, the metabolic viability was assessed using the Xpert Blue Cell Viability Assay (GRISP), following the manufacturer’s protocol. Resazurin reduction by viable cells was measured at Ex/Em (nm) 560/590 in a microplate reader (Synergy H11, BIOTEK, US). Relative viability was calculated in relation to the control of life and expressed graphically. Each sample was analyzed in duplicate in two independent assays.

### 2.12 Statistical Analysis

All measurements were carried out in triplicate, unless otherwise stated. Numerical data were reported as mean ± standard deviation (SD). Data were processed using GraphPad Software Prism 8.0 (GraphPad Software Inc., USA). The results were analyzed using One-way ANOVA and two-way ANOVA, Tukey and Sidak tests. Statistically significant differences were considered at *p* < 0.05.

## 3 Results and Discussion

### 3.1 Extraction of Bioactive Compounds

The extraction yields obtained for cork via method 1 and method 2, namely C1 and C2, were 18.51 ± 2,38 and 5.80 ± 0,91% respectively. The results indicate that the first extraction method, based on solvent renewal at each step, extracts bioactive compounds more efficiently than the second extraction method, based on replacing the extract at each extraction step but maintaining the same solvent throughout the process. These differences on extraction yield indicate a possible saturation (maximum extraction capacity) of the solvent used.

Nannan et al.^19^ performed cork extraction using the same equipment and program; however, they only carried out one extraction with 3 cycles, resulting in a yield of around 10%, a value lower than the obtained using method 1. Thus, replacing the used solvent in each step by a fresh one increases extraction yields, overcoming potential solvent saturation problems and ensuring that a greater amount of bioactive compounds are collected.

### 3.2 Biological Characterization of the Cork Extracts

Cork extracts were analyzed for their total phenolic compound (TPC) and total flavonoid compound (TFC) contents, as well as antioxidant and antibacterial activities. The TPC, expressed in gallic acid equivalents, is presented in Figure 1a. Note that a general assessment of the phenolics commonly associated with antioxidant properties in the extracts was carried out, and not a quantification of the complete phenolic profile. The analysis revealed that the extraction method significantly influences the TPC rate, reporting 315.79 ± 68.20 mg GAE/g for method 1 and 700.90 ± 96.31 mg GAE/g for method 2. Notably, the C2 extract exhibited the highest phenolic content, exceeding extraction rates from the literature using extraction method 2, namely 502 ± 25 mg GAE/g using the solvent H_2_O/EtOH (50:50 v/v).^20^ This improvement is explained by the replacement of the extract between cycles. Additionally, environmental factors such as light, temperature, nutrients, and water availability also influenced TPC.^21^ On its turn, the first method has been shown to report TPC for cork in between 200 and 250 mg GAE/g, with the value obtained for C1 being comparable.^22^

**Figure 1:**
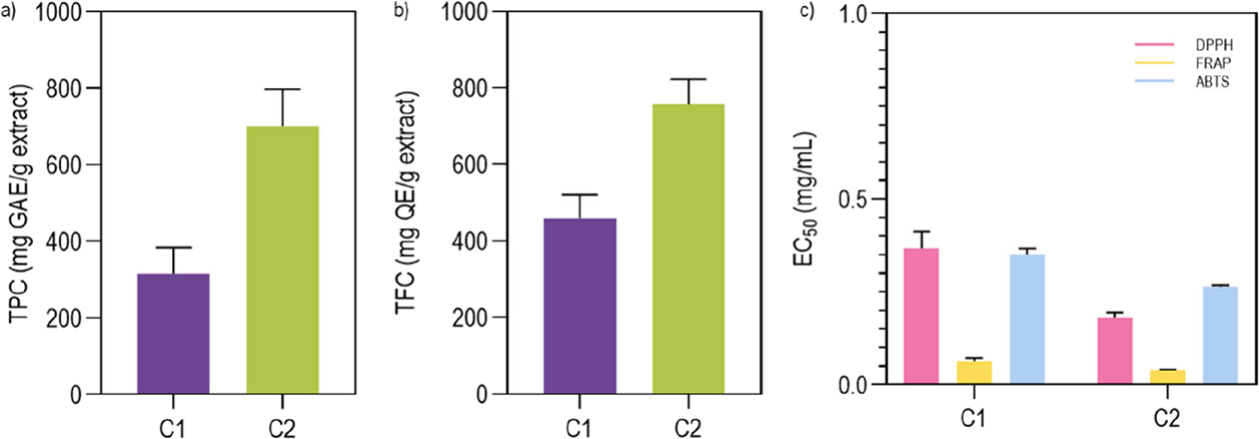
(a) TPC shown in gallic acid equivalents. Data are reported as mean ± SD (*n* = 2, where n corresponds to independent trials with 2 repetitions each); (b) TFC presented in quercetin equivalents. Data are reported as mean ± SD (*n* = 2, where n corresponds to independent trials with 3 replications each); (c) EC_50_ values (mg/mL) of all extracts under study for the different methods, DPPH, FRAP and ABTS. Data are presented as mean ± SD (*n* = 2, where n corresponds to independent trials with 3 replications each). Statistical significance was determined through multiple comparisons between the different methods using the Sidak test. Significance between DPPH vs FRAP, *p* < 0.0001; significance between DPPH vs ABTS, *p* < 0.01; and significance between FRAP vs ABTS, *p* < 0.0001.

The TFC in the extracts was determined and presented in quercetin equivalents (QE), as described in Figure 1b. The results demonstrated that, similar to TPC, TFC varies based on the extraction method applied. The TFC measured in compounds extracted by the first method (458.70 ± 61.76 mg EQ/g) was lower than that obtained using the second extraction method (755.93 ± 66.16 mg EQ/g).

The difference in total phenolic content (TPC) and total flavonoid content (TFC) between the extraction methods can be explained by the specific characteristics of each approach. In method 1, the solvent is renewed at the end of each extraction, whereas in method 2, the raw material is replaced. This means that, in the first method, the extract may undergo degradation due to prolonged exposure to the solvent. This continuous exposure can lead to the degradation of sensitive phenolic compounds or the loss of less stable compounds, resulting in a lower final TPC and TFC content. In contrast, in method 2, the constant renewal of raw material allows the extraction to occur in “fresher” substrates, preventing phenolic compounds and flavonoids from being exposed to the solvent for extended periods. As a result, the lower degradation of the extracted compounds may explain the higher TPC and TFC content observed in the extracts obtained by this method.^23^ Another factor to consider is the possible interaction between the hydroethanolic solvent and the extracted compounds. Depending on the exposure time, the solvent may promote the degradation of certain bioactive compounds or even alter their chemical conformation, reducing their final recovery.

The antioxidant activity of the obtained extracts was determined using three tests: DPPH, ABTS, and FRAP (Figure 1c). These assays explore different mechanisms of antioxidant performance, with DPPH, FRAP, and ABTS measuring activity via hydrogen atom transfer (HAT) and/or single electron transfer (SET): DPPH neutralizes radicals, FRAP reduces Fe^3+^ to Fe^2+^, and ABTS decolorizes the ABTS+ radical.^24^ The results were expressed in EC_50_ (mg/mL), which represents the extract concentration to capture 50% of the DPPH, ABTS, and FRAP radicals. Smaller EC_50_ values indicate stronger antioxidant activity. As expected, the antioxidant activity varied between extraction methods. Cork extracted by method 2 (C2) showed greater antioxidant activity, with an EC_50_ of 0.18 ± 0.01 mg/mL for DPPH, 0.04 ± 0.00 mg/mL for FRAP, and 0.26 ± 0.00 mg/mL for ABTS. A correlation was established between antioxidant capacity and TPC: those with the highest phenolic content promoted the greatest antioxidant activity due to the donation of hydrogen and electrons from the hydroxyl groups of these compounds.^25,26^

The antimicrobial properties of cork extracts C1 and C2 were evaluated by determining the minimum inhibitory concentration (MIC) and the minimum bactericidal concentration (MBC) against the Gram-positive bacterium *S. aureus* and the Gram-negative bacterium *P. aeruginosa*. The results are presented in Table 3.

**Table 3:** Minimal Inhibitory Concentration (MIC) and Minimum Bactericidal Concentration (MBC) of C1 and C2 Cork Extracts against *S. aureus* and *P. aeruginosa* Bacterial Cultures

sample	*S. aureus*		*P. aeruginosa*	
	MIC (mg/mL)	MBC (mg/mL)	MIC (mg/mL)	MBC (mg/mL)
C1			0.64	0.64
C2	0.16	0.08	0.32	0.32

C2 extract was the most effective against both bacteria, showing the highest activity against *S. aureus*. These results align with previous studies using other cork extracts.^27^ The observed differences can be attributed to variations in the composition of the cell wall of Gram-positive (*S. aureus*) and Gram-negative (*P. aeruginosa*) bacteria, as evidenced by their thickness and structural complexity. While Gram-negative bacteria have a more intricate structure, with a thin layer of peptidoglycans in close proximity to the cytoplasmic membrane and an outer lipopolysaccharide membrane that acts as a barrier, endowing them with greater resistance to antimicrobial agent penetration, Gram-positive bacteria have a wall composed of a dense layer of peptidoglycans.^28^ The structure of the cell wall of Gram-positive bacteria facilitates the entry of hydrophobic molecules, allowing interaction with both the cell wall and the cytoplasm. In contrast, Gram-negative bacteria possess an external lipopolysaccharide membrane that prevents the penetration of molecules, while periplasmic enzymes degrade those that manage to infiltrate.^29,30^

The C2 extract demonstrated inhibitory activity against both bacteria, which can be attributed to its high phenolic content (Figure 1), recognized for its antibacterial performance. Cork has hydroxyl groups that facilitate the formation of hydrogen bonds with proteins present in the bacterial membrane. This interaction alters the permeability of the membrane, making bacterial cells more susceptible to the extract’s action, including its penetration, which induces coagulation of the cellular contents and leads to the death of the organism.^29^ Considering the relationship between antimicrobial activity and phenolic content, the C1 extract, which has a lower phenolic content, exhibited reduced antimicrobial activity, with no effect observed against *S. aureus*.

Based on the analysis of Table 3, it was determined that the extract to be incorporated into the braids would be the C2 extract at a concentration of 0.32 mg/mL (MBC), a concentration capable of ensuring the elimination of both bacteria.

### 3.3 Assessment of the Braids’ Mechanical Properties

Mechanical tests were carried out to evaluate the most suitable conditions for producing artificial tendons from lyocell, PET and BP braids for potential uses in the replacement of human tendons. These trials explored the impact of different braid configurations on the overall mechanical performance of the artificial tendon, specifically focusing on the presence or absence of yarns at the core. Braids were produced with 16 threads in the sheath, either without a core (empty filling) or with a core composed of loose threads (4, 8, 16, or 32 threads) or smaller braids. This systematic approach aimed at evaluating how variations in core composition influenced the mechanical behavior of the structure. Based on the data collected (Section S1 in Supporting Information) and their proximity to the mechanical behavior required for human tendons repair or substitution,^2,3,20,31–33^ three braids were selected based (Table 4). The material that best met the application requirements was lyocell, with an elongation of less than 10% (≈8.72%) and strength at break between 15 and 100 MPa (average of 53.15 MPa).^2,3,31^ However, given that the objective was to combine the braids with a natural extract, which impact on the mechanical performance of the materials was unknown, none of the braids listed in Table 4 were excluded from further evaluations.

**Table 4:** Mechanical Performance of Lyocell, PET and BP Braids, Prepared with a Core Made of Loose Threads^a^

sample	elongation at break (%)	strength at break (MPa)
lyocell twist 14 *v*/″ – Braided exterior with *v* = 3.0 and core with 32 loose threads	8.72 ± 2.05	53.15 ± 12.62
PET – braided exterior with *v* = 3.0 and core with 32 loose threads	10.66 ± 5.04	44.12 ± 12.02
BP – braided exterior with *v* = 3.0 and core with 4 loose threads	18.72 ± 6.58	51.57 ± 15.91

a Results are presented as mean ± SD (*n* = 15; *v* = 3.0 represents the winding speed, while *v*/″ refers to turns per inch).

Although no literature could be found on lyocell braids, studies conducted on single, dried filaments estimated the polymer tensile strength to be around 1400 MPa and the elongation at break between 6 and 16%.^34,35^ One may predict a braid to have approximate characteristics, since it is a combination of filaments sharing the same properties; however, the distribution of forces in braids is closely related to the thickness of the yarn, the tightness of the braid and the presence or absence of a core, factors that can significantly influence these properties, as observed in Table 4 (strength at break).^36^

PET braids results revealed a significant discrepancy in relation to data reported in the literature. On average, the elongation and breaking strength of the engineered braids were 10.66% ± 5.04% and 44.12 ± 12.02 MPa, respectively. Yet, in the study by Debbabi et al., a braid of 16 PET yarns showed an elongation at break of 15.04 ± 0.24% and a maximum stress at break of 40.38 ± 0.16 N.^37^ The results of this study reported a maximum stress at break almost 4 times higher (Section S1 in Supporting Information). This discrepancy can be attributed to the presence of a core in the developed sample, composed of 32 loose strands, something that does not exist in the braid produced by Debbabi et al. The presence of the core increased tensile strength, as it provided structural support and facilitated load distribution between the strands. This finding was corroborated by the augment in strength at break registered as the number of strands in the braid increased (Section S1 in Supporting Information). Furthermore, differences in the form of production and possible variations in the composition of PET may also have influenced these variations between studies.

To date, there are no investigations dedicated to evaluating the mechanical performance of biodegradable polyester braids; as such, the results in Table 4 were compared to nonbiodegradable polyester braids. Zhang et al. developed a braid with 16 polyester threads, obtaining elongations at break between 10 and 24% and strength at break between 900 and 1300 N.^38^ Although the elongation at break was within the expected range, the maximum stress at break of the BP was lower than the reported (maximum stress at break of 293.27 ± 77.45 N, with a diameter of 2.71 ± 0.16 mm, and as such 51.57 ± 15.91 MPa). Differences in mechanical performance could be related to structural chemical changes between polymers. Even though the chemical composition of BP is confidential, it is expected this polymer to have been formulated to degrade more easily than nonbiodegradable polyesters. Consequently, the bonds between monomers and polymer chains are expected to be weaker. Additionally, the mechanical properties of BP braids may also be influenced by the manufacturing process, thickness of the threads, and characteristics of the actual braid (e.g., presence of a core).

### 3.4 Pretreatment of Braids

To eliminate the presence of potential residues or sizing on the yarns, the braids were washed three times in water at 500 rpm, at a temperature of 90 ± 5 °C for 30 min, and at a temperature of 60 ± 5 °C for another 30 min. The washing effect was verified by ATR-FTIR (Figure 2).

**Figure 2:**
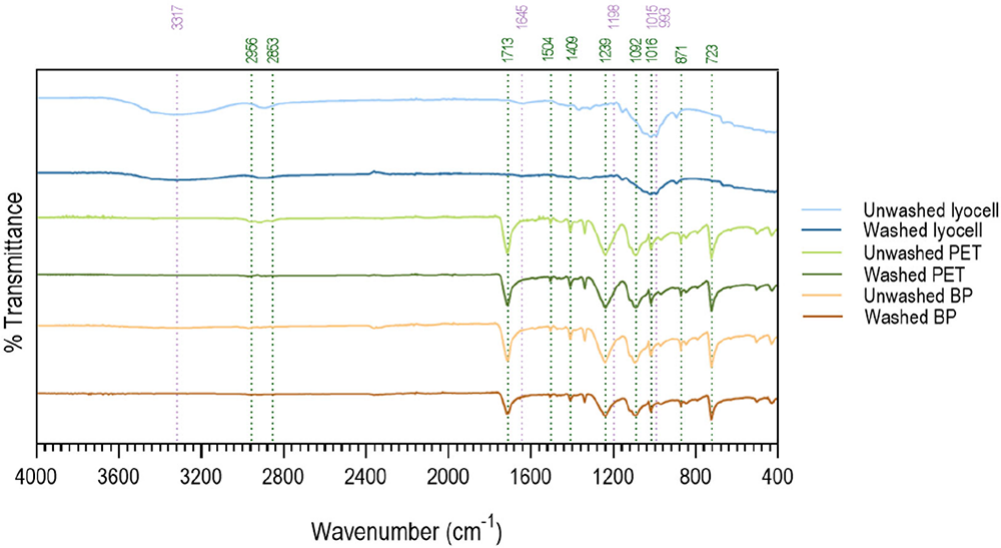
ATR-FTIR spectra of braids with and without washing collected between 400 and 4000 cm^–1^. Peaks associated with lyocell are identified in purple and peaks corresponding to PET and BP are highlighted in green.

Chemical groups characteristic of lyocell were detected by the infrared absorption band between 3000 and 3500 cm^–1^ and 1645 cm^–1^ associated with the stretching vibrations of the −OH group; at 1015 cm^–1^ associated with the asymmetric C–O–C stretching of NMMO; and at the spectral bands at 1198 and 993 cm^–1^ related to the N–O groups of NMMO. The remaining peaks are presented in Section S2 in Supporting Information.^39,40^ After washing, the characteristic peaks remained. The PET braid was confirmed by the peaks at 2956 and 2853 cm^–1^ attributed to stretching vibrations between carbon and hydrogen in the CH_2_ groups. PET registered a band as well at 1713 cm^–1^ referring to the C=O bond vibration of the carboxylic ester group, and the signals at 1579, 1504, and 1409 cm^–1^ attributed to the stretching of the aromatic skeleton. In the PET spectrum, the bands at 1454 and 871 cm^–1^ correspond to CH_2_ bending and CH_2_ oscillation, respectively. Additionally, the bands at 1239 and 1092 cm^–1^ are associated with the stretching of the C–C–O bond in the ester group, while the peaks at 1016 and 723 cm^–1^ are related to the in-plane C–H stretching and out-of-plane C–H bending of the aromatic ring, respectively.^41^ BP is made of terephthalate acid and ethylene glycol, the same elements present in PET and, as such, the peaks found in BP can be equated to those of PET. Between the two polymers, only slight differences were observed, which may be related to specific bonds or chemical structures responsible for the biodegradable quality of BP. Both in PET and BP, no differences were identified between the braids before and after washing; again, the characteristic peaks remained in the same regions.

To evaluate whether the washing process influenced the thermal stability of the material, TGA analyzes were performed. This technique allows measuring the variation in sample mass as a function of increasing temperature in a controlled environment (Figure 2).

Figure 3 shows the thermograms for (a) lyocell, (b) PET, and (c) BP, before and after washing. In all materials, an overlap of the mass variation curves with temperature is noted, as well as a mass loss above 80% at 600 °C. The results indicate that the presence of potential impurities in the braids has no effect on the thermal properties of the polymers.

**Figure 3:**
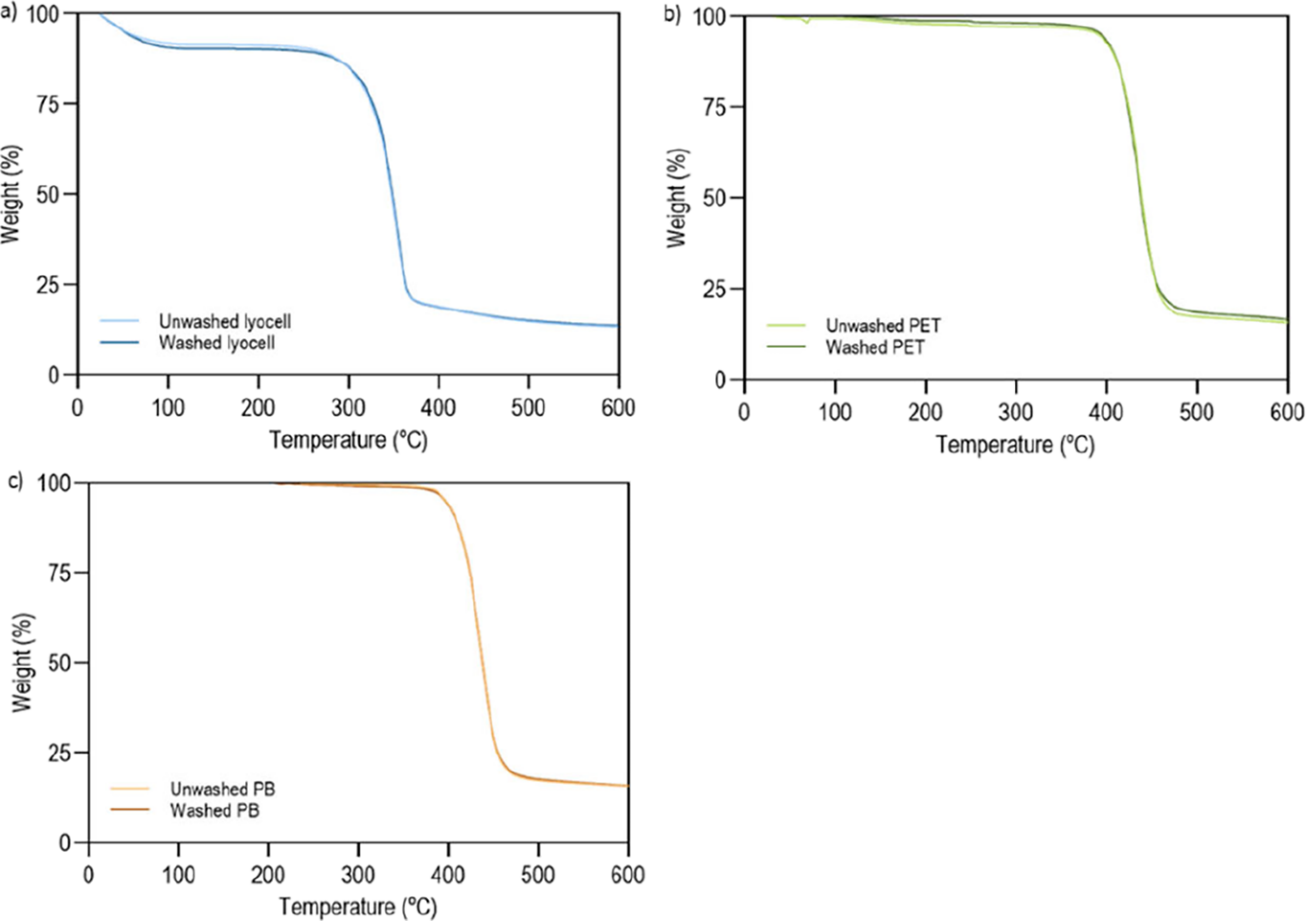
TGA thermograms of (a) lyocell, (b) PET and (c) BP, washed and unwashed, obtained under a nitrogen atmosphere of 200 mL/min.

Pre- and postwash lyocell (Figure 3a) undergoes an abrupt decrease in mass at approximately 300 °C. When the temperature reaches 379 °C the mass loss becomes less important. At 600 °C, only about 13.8% carbon residue remains. During combustion, phosphorus components release polyphosphoric acids, phosphoric acids and other phosphorus-containing derivatives, facilitating the dehydration of cellulose and the formation of a greater amount of residue.^42^

The thermograms for PET and BP samples before and after washing (Figure 3b,c) are identical, observing the presence of residues in the order of 16.6% once reaching 600 °C. In these polymers, the main mass loss starts at around 400 °C, slowing down around 470 °C. Here, polymer degradation begins with the decomposition of the secondary chains and progresses to the polymer main chain.^43^

It is also important to note that among the 3 polymers tested, lyocell is the one that appears to have the greatest affinity with water, since the first reduction in mass detected for this braid begins at around 45 °C with the evaporation of water molecules attached to the fibers.

Mechanical analyzes of unwashed and wet (immersed in PBS), unwashed and dried, washed and dried, and washed and wet (immersed in PBS) braids were carried out to understand the effect of washing and the presence of moisture on the braids mechanical performance (Table 5).

**Table 5:** Mechanical Properties up to Failure of Unwashed, Washed, Dried and/or Wet Lyocell, PET and BP Braids^a^

sample	unwashed + wet		washed + wet		unwashed + dry		washed + dry	
	elongation at break (%)	strength at break (MPa)	elongation at break (%)	strength at break (MPa)	elongation at break (%)	strength at break (MPa)	elongation at break (%)	strength at break (MPa)
lyocell	14.55 ± 1.55	30.10 ± 4.29	13.05 ± 1.35	35.29 ± 7.39	8.72 ± 2.05	53.15 ± 12.62	9.92 ± 1.30	37.29 ± 6.47
PET	12.77 ± 0.99	55.81 ± 11.37	15.70 ± 0.82	44.03 ± 6.67	10.66 ± 5.04	44.12 ± 12.02	14.35 ± 1.66	35.92 ± 6.49
BP	15.71 ± 2.34	41.00 ± 8.17	16.37 ± 1.61	41.30 ± 9.04	18.72 ± 6.58	51.57 ± 15.91	16.76 ± 2.15	40.34 ± 6.74

a Results are presented as mean ± SD (*n* = 15).

In polymers such as lyocell, the higher elongation observed in wet environments can be attributed to the plasticizing effect of water, which reduces interchain interactions (such as hydrogen bonds), increasing their mobility. Conversely, the decrease in tensile strength under wet conditions, particularly when the braids are unwashed, can be explained by chain reorganization and a potential loss of structural cohesion.^34,44^ However, when the braids are washed, the strength at break remains practically unchanged, suggesting that the washing process may remove coatings or surface residues that could compromise mechanical strength.

For PET, which has a more hydrophobic and stable molecular structure, the literature often reports superior mechanical properties under dry conditions.^45^ However, the absence of this difference in the present results may be related to the use of braids with a core, where the interaction between the core and the shell, as well as the entanglement of the fibers, generates a more compact structure that is less sensitive to moisture. Additionally, the presence of a potential textile coating on the yarns, applied to facilitate their movement during weaving, may influence the results in terms of both strength and elongation. While this effect is relevant to produce fabrics or knitted textiles, it may be less pronounced in braids due to their tight formation (organizational structure) and the reduced mobility of the fibers.

In the case of BP, the results indicate that braids in the dry state exhibit greater flexibility compared to those in the wet state. This behavior can be explained by the increased stiffness induced by moisture, which reduces internal friction between the fibers and limits deformation.

Overall, the impact of moisture on polymers may depend on their chemical composition and interaction with coatings, if present.

### 3.5 Braids’ Functionalization

The functionalization of the braids was carried out through physical absorption. This is a simple process based on sample immersion in a solution containing the active agent, and is favored by the variety of interaction forces promoted, including hydrophobic bonds, electrostatic bonds, hydrogen bonds or van der Waals forces.^46^

Various C2 extract concentrations (MBC, 2 × MBC, 5 × MBC and 10 × MBC) were prepared, and the braided structures were immersed for 24 h. The amount of C2 extract retained by the braids, after 3 consecutive washings to eliminate weakly bound molecules, is shown in Table 6. Based on the results, 5 × MBC concentration was selected for the subsequent functionalization of the braids, as it was the least concentrated solution (for braid immersion) from which it was possible to achieve a sample loading superior to the highest MBC (Table 3) in all samples. Since 0.32 mg/mL (MBC) of C2 extract was found to be the minimal concentration required for eliminating both bacteria, all subsequent testing with functionalized braids was based on this premise.

**Table 6:** Amount of C2 Extract Loaded onto Lyocell, PET and BP Braids via Physical Adsorption According to the Concentrations of Immersing Solution (MBC, 0.32 mg/mL; 2 × MBC, 0.64 mg/mL; 5 × MBC, 1.60 mg/mL; 10 × MBC, 3.20 mg/mL)^a^

sample	MBC (mg/mL)	2 × MBC (mg/mL)	5 × MBC (mg/mL)	10 × MBC (mg/mL)
lyocell	0.23 ± 0.04	0.25 ± 0.05	0.55 ± 0.14	1.20 ± 0.34
PET	0.19 ± 0.02	0.18 ± 0.05	0.45 ± 0.20	0.76 ± 0.25
BP	0.12 ± 0.01	0.17 ± 0.02	0.33 ± 0.16	0.73 ± 0.36

a Results are presented as mean ± SD (*n* = 3).

The extract loading results in Table 6 show some variability, especially in BP and PET braids. This heterogeneity likely stems from differences in surface chemistry, porosity, hydrophilicity, and minor inconsistencies during braid fabrication. These factors affect the adsorption efficiency of the cork extract (C2). Although physical adsorption was selected for its simplicity and biocompatibility, we recognize its limitations in reproducibility. Future work will focus on optimizing uniformity through plasma surface treatment followed by dip coating, as well as exploring encapsulation strategies to improve extract retention and consistency.

### 3.6 Characterization of Braids Pre and Post Functionalization

#### 3.6.1 Morphology

Figure 4 shows the morphological aspect of the pre- and postfunctionalized braids once tension is applied at the extremities to display the core (4.2× magnification). After functionalization, the lyocell braid presented a more intense brown color, indicating a greater amount of C2 extract functionalized. By applying compression strength on the extremities of the samples (as represented by the tweezers), it was possible to visualize the core of the lyocell and BP braids. This was not accessible on PET braids due to their tighter structure.

**Figure 4:**
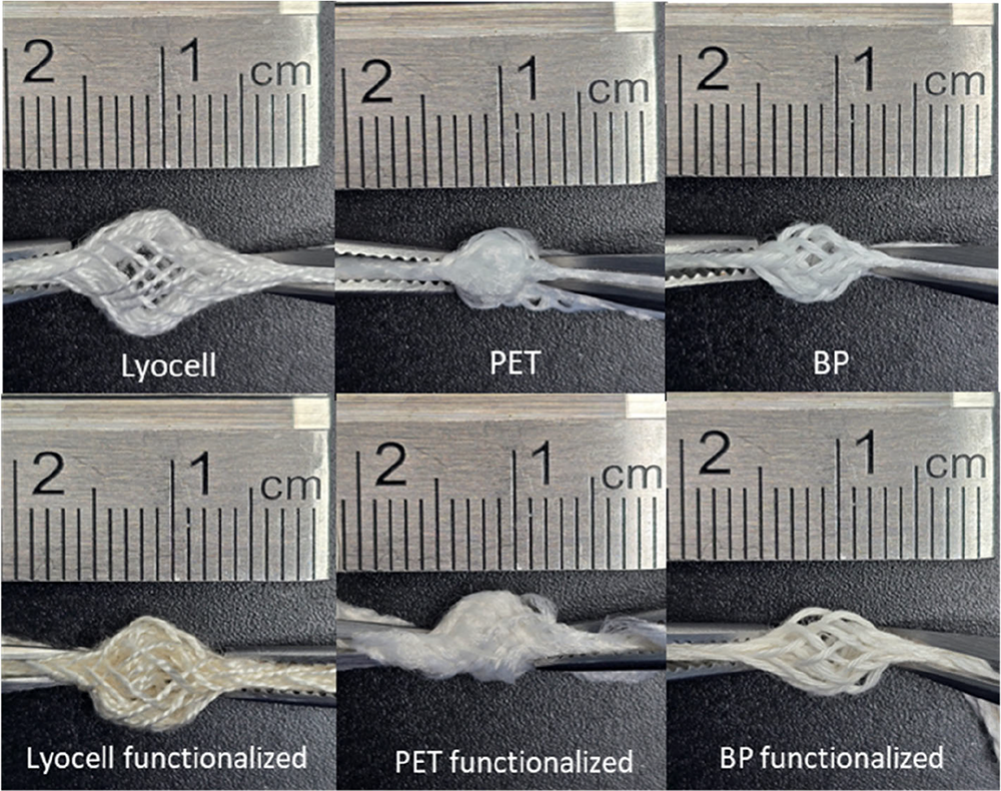
Lyocell, PET and BP braids before and after functionalization with C2, captured with a Samsung Galaxy S24 Ultra smartphone cell phone camera at 9 cm distance from the braid and 4.2× magnification.

The diameter of the braids was determined before and after functionalization in dry and wet states (Table 7). Small fluctuations in diameters were observed within the same braid typology, which are considered inevitable since production and collection methods cannot be entirely replicated between braids (tensions are initially dependent on the strength of the user; there is no automatization for pulling the yarns through the equipment set up). These variations also result from differences in the opening of the braided yarns (between yarns).

**Table 7:** Diameter of Lyocell, PET and BP Braids, before and after Functionalization, in Dry or Wet States (Immersed in PBS), Determined Using a Caliper^a^

sample	diameter before functionalization (mm)		diameter after functionalization (mm)	
	dry state	wet state	dry state	wet state
lyocell	3.35 ± 0.17	3.43 ± 0.29	3.41 ± 0.16	3.60 ± 0.12
PET	2.49 ± 0.12	2.70 ± 0.20	2.92 ± 0.22	2.65 ± 0.18
BP	2.71 ± 0.16	2.71 ± 0.24	2.66 ± 0.18	2.40 ± 0.06

a Results were presented as mean ± SD (*n* = 15).

After washing and drying, an increase in diameter was observed and, consequently, a decrease in tensile strength at failure (Table 5); however, such reduction was not considered problematic since elongation remained less than 10% and tensile strength was between the accepted range for hand tendons, there is 15 and 100 MPa.^2,3,31^ Among the testing subjects, the lyocell braid was deemed the closest to the desired range for hand tendons replacement/substitution, namely between 4.0 ± 0.7 and 4.7 ± 0.7 mm,^7^ since after functionalization the diameter for the dry and wet braids was of 3.41 ± 0.16 and 3.60 ± 0.12 mm, respectively.

No correlation was found between the increase in diameter and the state of the braids, dry or wet. Likewise, no relationship was observed between the diameter and the presence of cork extract in braids. As stated earlier, small fluctuations among samples of the same typology are inevitable.

#### 3.6.2 ATR-FTIR

ATR-FTIR spectra (Figure 5) were collected to confirm the presence of the C2 extract after braids’ functionalization. Cork extract has an infrared absorption band at 3294 cm^–1^, associated with the stretching of hydroxyl groups. Peaks were also identified at 2927 and 2850 cm^–1^, attributed to the suberin aliphatic chains, characteristic of asymmetric and symmetric C–H stretching vibrations. The absorbance peaks at 1716, 1316, and 1175 cm^–1^ were attributed to C=O and the symmetric and asymmetric C–O stretching of the suberin ester group, respectively. The peak recorded at 1447 cm^–1^ is characteristic of the asymmetric C–H deformation of suberin, as well as lignin and polysaccharides. The C=C stretching recorded at 1606 and 1505 cm^–1^ was attributed to vibrations of the aromatic ring of G-lignin. The absorbance peaks at 1095 and 1035 cm^–1^ are characteristic of the C–O stretching vibrations of polysaccharides and lignin on cork, respectively.^47^

**Figure 5:**
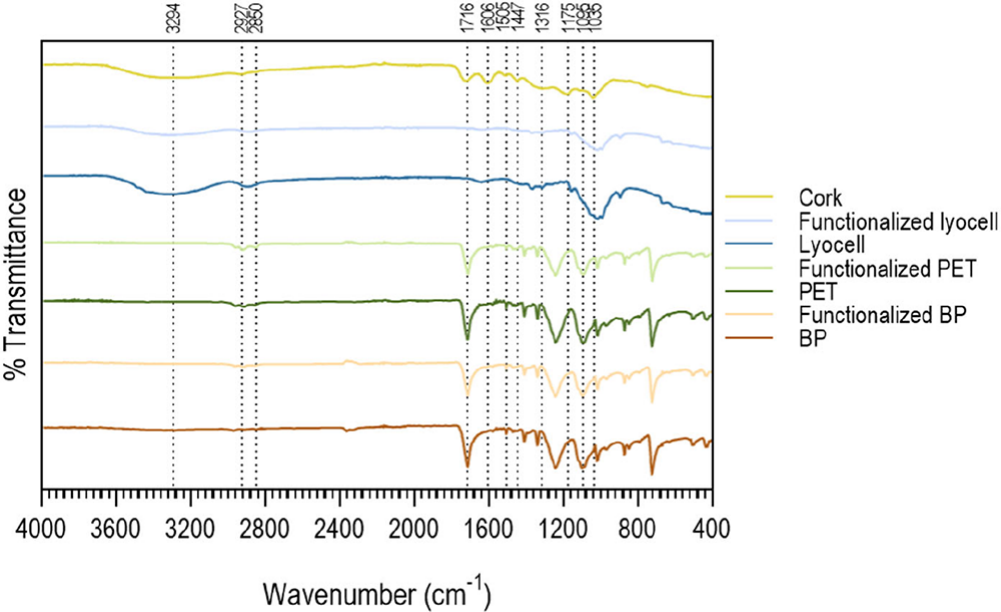
ATR- FTIR spectrum of cork extract and the polymeric braids made of lyocell, PET and BP, before and after functionalization.

By analyzing Figure 5, it was evident that for all braids, after functionalization, the peaks corresponding to each polymer became smaller (discussed in Section 3.4), this being more visible for the lyocell braid. This behavior is consistent with the morphological analysis (Figure 4), which showed a more intense brown color for the lyocell braid, indicating a higher amount of functionalized C2 extract. Furthermore, the loading results (Table 6) confirmed that lyocell exhibited the highest retention capacity of the active agent, suggesting that its structure allows for better absorption and retention compared to BP and PET braids. Although the presence of cork was not detected on the braids due to its low loading concentration, possible chemical interactions, such as hydrogen bonding and van der Waals forces, may have contributed to these small differences. These interactions likely occurred between the functional groups of the cork extract (e.g., hydroxyl and carboxyl groups) and the polymeric chains of the braids, enhancing the adhesion and retention of the extract.

#### 3.6.3 Thermal Characterization

To evaluate the thermal behavior of the braids, before and after functionalization with C2 extract, two analyses were carried out, TGA and DSC (Figure 6). The TGA analysis of the cork extract (control) can be found in Figure 6Id.

**Figure 6:**
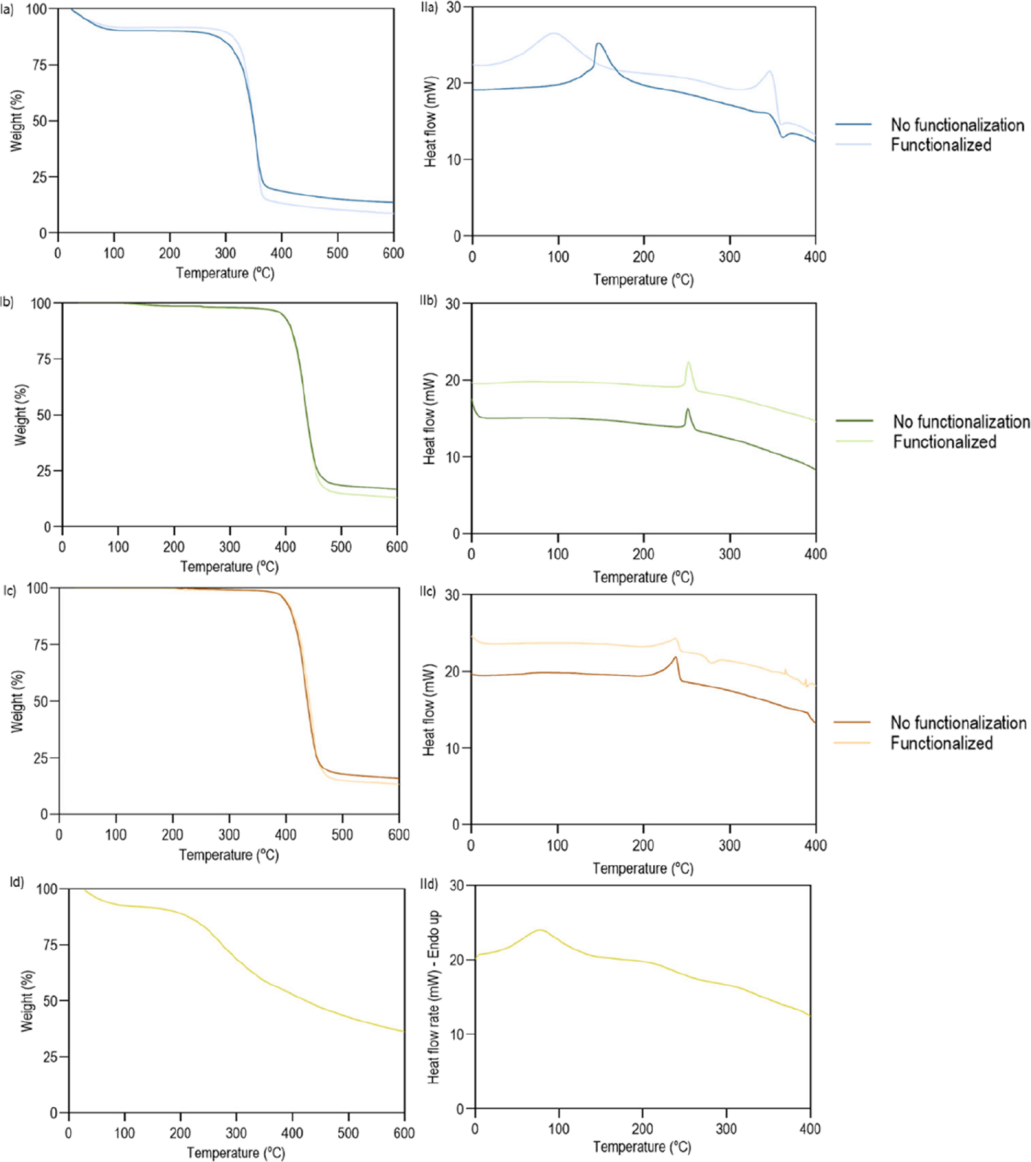
(Left) Thermograms obtained by TGA of braids of (Ia) lyocell, (Ib) PET, (Ic) BP, and (Id) C2 extract, pre and post functionalization, obtained under a nitrogen atmosphere at 200 mL/min. (Right) Thermograms obtained by DSC of the braids (IIa) lyocell, (IIb) PET, (IIc) BP, and (IId) C2 extract, pre and post functionalization, obtained under a nitrogen atmosphere at 20 mL/min.

In the TGA analyzes of lyocell (Figure 6Ia), an initial mass loss of around 10% was observed, corresponding to the volatilization of residual moisture.^48^ Lyocell without C2 extract (Figure 6Ia) exhibited a sudden decrease in mass between ≈290, and 379 °C. At 600 °C, only around 13.8% of the mass remained, revealing the typical behavior of this material.^42^ After functionalization, a sharp mass loss occurred at ≈310 °C, and at 600 °C, only ≈8.7% of carbon char remained. These results indicate a slight influence of cork on the thermal properties of the lyocell, leading degradation to a higher temperature, around 310 °C. The reduction in the amount of residues identified at 600 °C may be associated with an additional decomposition of the polymer and extract induced by the bonds established between the two parts (such as hydrogen bonding, van der Waals forces, and possible covalent interactions between hydroxyl groups of lyocell and functional groups present in the cork extract).

Regarding the TGAs of PET and BP (Figure 6Ib), the thermal response was found to be very similar across samples, regardless of the presence of the functionalized C2 extract. The most significant degradation occurred between 400 and 470 °C, with the decomposition of the secondary and main polymer chains, leaving only 16.6% of mass at 600 °C. This behavior is characteristic of these materials, confirming that BP exhibited thermal stability comparable to PET.^49,50^ Similar to the functionalized lyocell braids, the presence of C2 extract led to a reduction on the carbon chair reduced (13.1%). These results suggest that the initial thermal stability of the polymer remains unaffected, while the interactions between braid and extract (such as hydrogen bonding, van der Waals forces, and possible dipole–dipole interactions) may contribute to higher materials’ decomposition at 600 °C.

DSC thermograms of lyocell, PET and BP braids, with and without functionalization, are reported in Figure 6II (DSC for C2 extract is shown in Figure 6IId).

The melting temperature (*T*_m_), degradation temperature (*T*_d_) and enthalpy (Δ*H*) were determined for all samples (Table 8). *T*_m_ represents the temperature at which a phase change is observed, *T*_d_ represents the point at which the material begins to degrade, and enthalpy is an indicative of the amount of energy absorbed during thermal transitions.^50,51^

**Table 8:** Δ*H*, *T*_m_, Start Temperature (*T*_o_) and *T*_d_ of the Lyocell, PET and BP Braids before and after Functionalization with C2 Extract

sample	Δ*H* (J/g)	*T*_m_ (°C)	*T*_o_ (°C)	Δ*H* (J/g)	*T*_d_ (°C)	*T*_o_ (°C)
lyocell	98.40	146,88	139.95	–21.82	360.89	351.52
functionalized lyocell	109.90	94.07	56.43	58.48	346.78	327.40
PET	30.29	251.11	247.40			
functionalized PET	35.13	251.93	247.20			
BP	32.64	237.09	221.79			
functionalized BP	32.89	236.76	220.53			

Variations in the thermal behavior of lyocell indicate alterations in its potential physical properties and molecular structure before and after functionalization with C2. As shown in Figure 6IIa, the DSC analysis of lyocell braids reveals an exothermic peak at 146 °C for the nonfunctionalized sample, corresponding to its melting temperature (*T*_m_). After functionalization, the Tm decreased to 94.07 °C, suggesting that the material may have undergone structural modifications that facilitate phase transitions.^52^ The enthalpy (Δ*H*) of the functionalized lyocell was higher (109.90 J/g) compared to the nonfunctionalized sample (98.40 J/g), indicating that the functionalized lyocell requires more energy for phase transition, which may result from interactions between the lyocell and the cork extract. Additionally, a second thermal event associated with polymer degradation was observed around 350 °C, which aligns with the TGA results in Figure 6Ia, where a significant mass loss occurs at this temperature range. This degradation behavior confirms that functionalization affects the thermal stability of the material. Overall, the results suggest that functionalized lyocell is more easily processable due to possible chemical changes in its structure, making it more susceptible to thermal transitions and degradation at lower temperatures.

Modifications with C2 extract did not alter the thermal properties of PET, with the melting temperature being detected at ≈251 °C in both samples.^53^ The same happened with BP, denoting similar thermal characteristics between the two braids under study (PET vs BP), regardless of functionalization with extract. For both PET and BP, functionalization had a minimal impact on the thermal properties of the materials, as observed from the TGA results (Figure 6Ib,Ic). For these materials, it was not possible to reach the degradation temperature, as it tends to occur at temperatures superior to 400 °C (Figure 6II), which surpassed equipment sensitivity.

Data demonstrated that lyocell braids exhibit the greatest affinity toward the extract, establishing potential chemical bonds, in addition to physical ones, and, thus, altering the thermal response of the polymer. In turn, PET and BP braids appeared to establish only physical connections with C2 extract, with a negligible influence on the polymers’ thermal response.

#### 3.6.4 Wettability and Surface Free Energy

The wettability of the surface of the braids, before and after C2 extract functionalization, was measured by water contact angle determinations. Based on these measurements, surfaces can be classified into four categories: superhydrophilic surfaces, with an angle of less than 5°, where the droplet is absorbed the moment it comes into contact with the surface; hydrophilic surfaces, with a contact angle of less than 90°, which are easily wetted; hydrophobic surfaces, with a contact angle equal to or greater than 90°, which are moderately resistant to water; and superhydrophobic surfaces, with a contact angle greater than or equal to 150°, which have high water resistance.^54,55^

Table 9 presents data on the behavior of the braids when in contact with water. Lyocell braids exhibited superhydrophilic behavior, demonstrating uniform moisture absorption. On the other hand, the PET braid exhibited hydrophobic behavior, which was reduced by around 23° after cork functionalization.^56^ BP braids showed superhydrophobic behavior, which shifted toward a hydrophobic nature after cork loading (reduction in ≈5°).^57^ It is important to highlight that contact angles were measured on woven materials, which have porosities and, consequently, may facilitate water penetration, influencing wettability determinations.

**Table 9:**
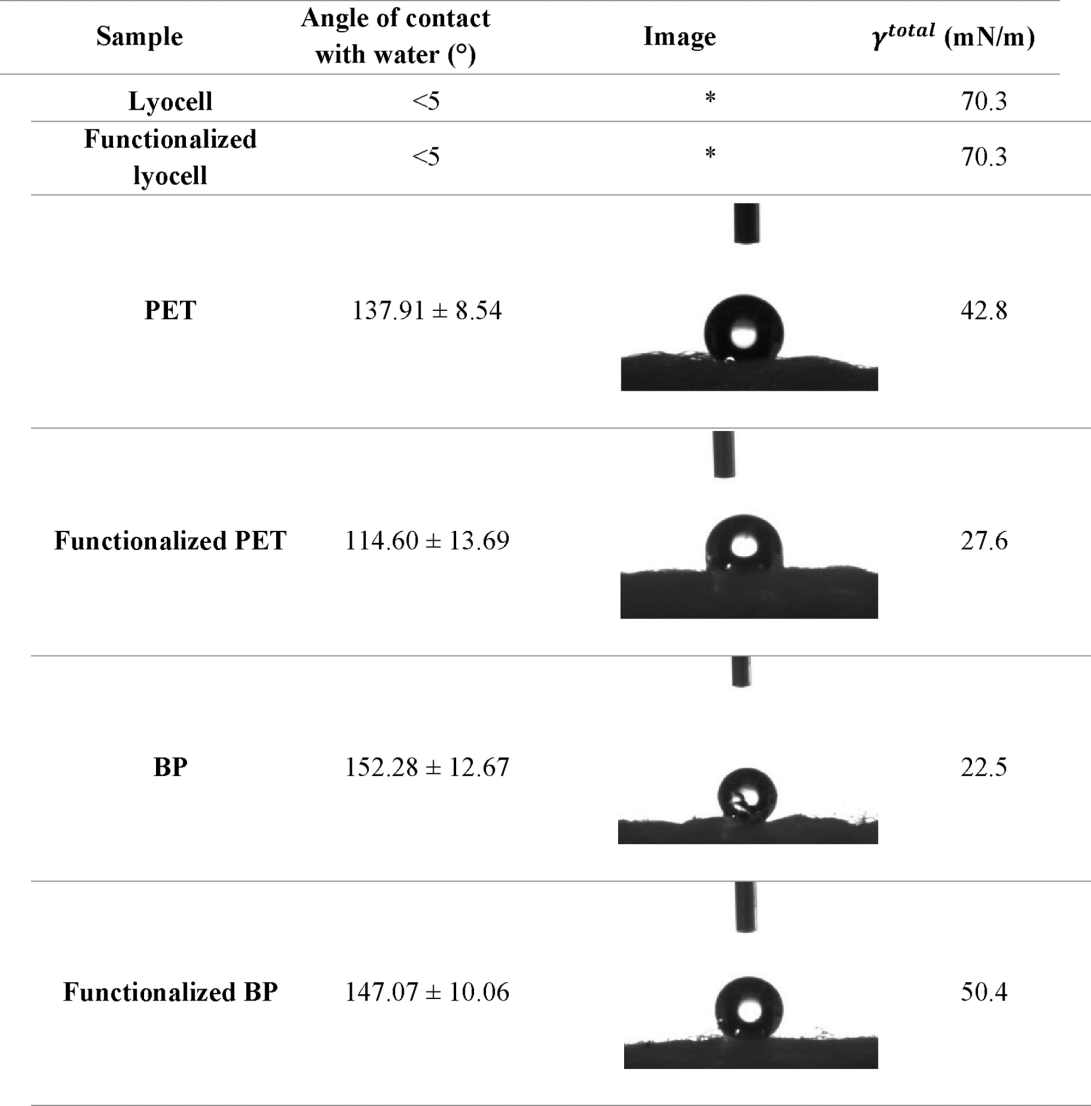
Determination of the Water Contact Angle and SFE (*n* = 5) of Lyocell, Functionalized Lyocell, PET, Functionalized PET and BP Samples

a Droplet completely absorbed by the substrate after contact, making it impossible to capture images.

Surface free energy (SFE) was calculated using the Owens and Wendt method, which divides the total surface energy of materials into polar and dispersive (nonpolar) components, thus providing a more in-depth understanding of the properties of braids and their capacity of interact with the bioactive agent (Table 9). This energy is determined by measuring the contact angles with different liquids (Section S4 in Supporting Information), namely ultrapure water (polar), ethylene glycol (nonpolar) and diiodomethane (nonpolar), which have low volatility and do not swell the braids.

Generally, surfaces with higher SFE exhibit lower contact angle and greater wettability. They are also known to promote strong molecular attraction, making it easier to establish connections with the surrounding environment.^18^ The lyocell braid, both functionalized and nonfunctionalized, was categorized as superhydrophilic and obtained an SFE of 70.3 mN/m, while the nonfunctionalized and functionalized BP, presented an SFE of 22.5 and 50.4 mN/m, respectively. On the other hand, PET braids without extract obtained an SFE of 42.8 mN/m and the functionalized 27.6 mN/m. Contrary to expectations, the nonfunctionalized PET, which was the most hydrophobic surface, exhibited a higher SFE compared to the functionalized PET. These results suggest heterogeneity in the functionalization process, indicating that the binding mechanism with this substrate may be different from that of the other polymers (Table 9).

#### 3.6.5 Hydration and Water Retention

The hydration and water retention of the braids was determined by measuring weight variations between dry samples and after incubation in 0.1 M PBS (pH 7.4) at 37 °C for up to 7 days (Table 10). Braids were weighed at various time intervals (2, 4, 6, 24, 48, 72, and 168 h) to identify the hydration balance. An initial hydration greater than 40% was observed for all samples. During 168 h slight fluctuations in weight were observed, but less than 15%. PET samples registered the highest degree of hydration (69.26 ± 2.28% was the highest for bare PET and required 24 h of incubation, while the highest for functionalized PET was reached after 2 h and it was 63.69 ± 4.14%). For PET, it was also observed that weight remained practically unchanged between 48 and 72 h, and between 6 and 24 h for functionalized PET. Regarding water retention (WR), it is observed that lyocell and PET achieved the greatest percentages of WR, with values higher than 200%.

**Table 10:** Hydration and Water Retention of the Different Braids, Pre and Post Functionalization, after Immersion in PBS for 2, 4, 6, 24, 48, 72, and 168 h, Incubated at 37 °C (*n* = 3)

sample	hydration (%)						
	2 h	4 h	6 h	24 h	48 h	72 h	168 h
lyocell	56.57 ± 3.37	53.77 ± 3.05	57.56 ± 2.92	57.40 ± 0.42	59.27 ± 1.49	56.78 ± 0.95	56.28 ± 1.34
functionalized lyocell	57.40 ± 4.16	53.96 ± 6.60	57.24 ± 5.30	58.19 ± 2.36	58.37 ± 2.86	57.85 ± 2.76	56.46 ± 4.29
PET	59.06 ± 8.98	55.41 ± 8.65	62.58 ± 2.19	69.26 ± 2.28	66.83 ± 1.72	66.27 ± 5.50	58.40 ± 5.41
functionalized PET	63.69 ± 4.14	59.47 ± 6.34	57.40 ± 2.51	57.82 ± 4.84	55.38 ± 1.69	53.10 ± 6.91	50.43 ± 7.02
BP	42.90 ± 8.16	36.02 ± 8.35	40.43 ± 10.82	47.00 ± 9.02	44.88 ± 8.37	44.16 ± 10.81	43.14 ± 11.48
functionalized BP	46.32 ± 6.35	39.59 ± 7.05	41.73 ± 2.87	47.59 ± 7.14	47.32 ± 5.58	45.49 ± 1.94	48.70 ± 3.49
sample	water retention (%)						
	2 h	4 h	6 h	24 h	48 h	72 h	168 h
lyocell	231.22 ± 18.69	216.90 ± 13.85	236.28 ± 15.64	234.77 ± 2.33	245.75 ± 8.84	231.43 ± 5.01	228.85 ± 6.94
functionalized lyocell	236.28 ± 23.96	220.43 ± 33.86	236.48 ± 31.43	239.69 ± 13.84	240.95 ± 16.72	237.94 ± 16.14	231.13 ± 22.21
PET	251.42 ± 49.03	229.48 ± 40.31	267.84 ± 15.96	326.47 ± 23.49	302.01 ± 15.60	301.68 ± 48.26	243.05 ± 30.96
functionalized PET	277.92 ± 32.85	251.17 ± 42.48	235.28 ± 13.66	239.13 ± 26.91	234.45 ± 59.70	216.56 ± 34.23	204.68 ± 31.31
BP	177.37 ± 23.52	157.99 ± 19.51	171.30 ± 28.37	192.16 ± 30.66	184.02 ± 25.76	183.43 ± 33.89	180.33 ± 33.04
functionalized BP	187.97 ± 21.40	166.96 ± 18.41	171.88 ± 8.48	193.04 ± 24.66	191.16 ± 19.08	183.62 ± 6.66	195.51 ± 12.83

The absorption of water by different materials is related to wettability, the material’s chemical structure and the capillary forces of the fibers.^58,59^ The percentage of hydration is an important factor in controlling the release rate of active agents. The greater the percentage of hydration, the easier the release to the desired environment occurs.^59^ Because lyocell has a high percentage of fibrillar structures, water molecules diffuse more easily.^58^ It is important to note that braids are formed by a substantial group of fibers, and as such water molecules have a large surface area available to establish interactions.

It is important to note that the results obtained were influenced by the presence of salts in PBS, which can interact with the braided fibers, altering their hydration properties. These interactions may occur through ionic or hydrogen bonds between the salts and the fibers, and despite subsequent washings, there seems to be an effect on the water absorption and retention capacity of the materials studied.

#### 3.6.6 Porosity

Porosity is crucial to the performance of the braid as a tendon, as it facilitates cell adhesion, the exchange of nutrients and oxygen, and promotes vascularization, generating an environment conducive to matrix regeneration and cell proliferation.^60,61^ In this way, porosity was determined by varying the weight of the sample after immersion in ethanol for 1 h. The results are reported in Table 11, where it is possible to verify that after functionalization, for all samples, the porosity increases slightly, thus being favorable for the intended application. The order of magnitude for the porosity is similar across materials; however, more significant differences can be observed for PET, which once again may reflect the heterogeneity previously discussed in Section 3.6.4. This increase in porosity may be related to the dynamic environment in which C2 extract functionalization occurs and also to potential structural changes induced in the fibers after extract binding. These structural changes could involve potential electrostatic repulsions and should be considered alongside the possible types of bonds formed during the interaction.

**Table 11:** Braids’ Porosity before and after C2 Extract Functionalization (*n* = 3)

sample	porosity (%)
lyocell	20.61 ± 4.15
functionalized lyocell	22.25 ± 0.51
PET	17.32 ± 2.72
functionalized PET	24.51 ± 3.12
BP	20.03 ± 1.05
functionalized BP	20.58 ± 3.26

#### 3.6.7 Mechanical Examinations of Functionalized Braids

Tensile tests were carried out to determine whether the mechanical properties of the braids were influenced by the presence of C2 extract (Table 12). After analysis and taking into account the results obtained for braids without functionalization, no major differences were observed, and as such, the addition of the natural extract was deemed unimpactful on the mechanical characteristics of the polymeric braids (as discussed in Section 3.4).

**Table 12:** Mechanical Properties of Lyocell, PET and BP Braids, Pre and Post Functionalized, Up to Failure^a^

sample	wet		dry	
	elongation at break (%)	strength at break (MPa)	elongation at break (%)	strength at break (MPa)
lyocell	13.05 ± 1.35	35.29 ± 7.39	9.92 ± 1.30	37.29 ± 6.47
functionalized lyocell	15.26 ± 0.68	34.25 ± 3.83	10.80 ± 0.85	35.43 ± 4.14
PET	15.70 ± 0.82	44.03 ± 6.67	14.35 ± 1.66	35.92 ± 6.49
functionalized PET	16.50 ± 1.08	45.35 ± 5.15	15.38 ± 2.32	41.37 ± 6.68
BP	16.37 ± 1.61	41.30 ± 9.04	16.76 ± 2.15	40.34 ± 6.74
functionalized BP	16.76 ± 1.96	52.45 ± 6.75	16.83 ± 1.63	52.26 ± 7.20

a Results presented as mean ± SD (*n* = 5).

#### 3.6.8 Degradation in Physiological-like Media

The stability of the braids in a physiological environment was measured over a three-month period, based on mass variation (Figure 7), tensile strength (Figure 8) and thermal response (TGA; Figure 9). Due to the low concentration of loaded cork in the braids and the lack of influence on the thermal behavior (Section 3.6.3) and mechanical performance (Section 3.6.7), only pristine, washed braids were examined for their degradation profile overtime. Testing required immersing 5-m braids in 1 L of PBS (pH 7.4) at 37 °C.

**Figure 7:**
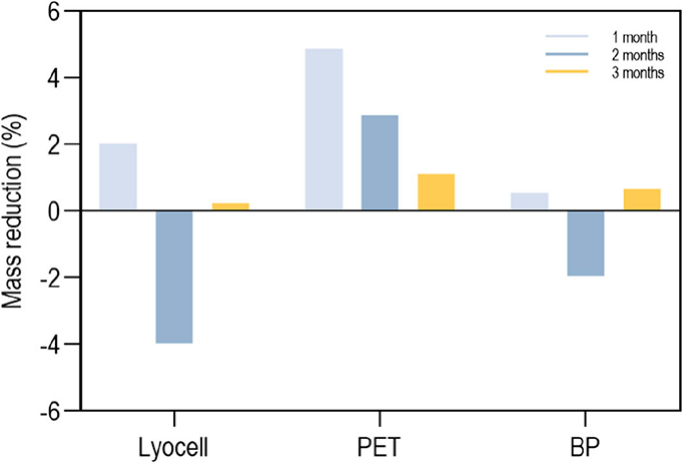
Braid degradation profile (mass reduction) for 3 months in 0.1 M PBS (pH 7.4) at 37 °C. Data correspond to a single continuous braid for each polymer. The subdivision into testing samples (smaller dimensions) was carried out after mass reduction examinations.

**Figure 8:**
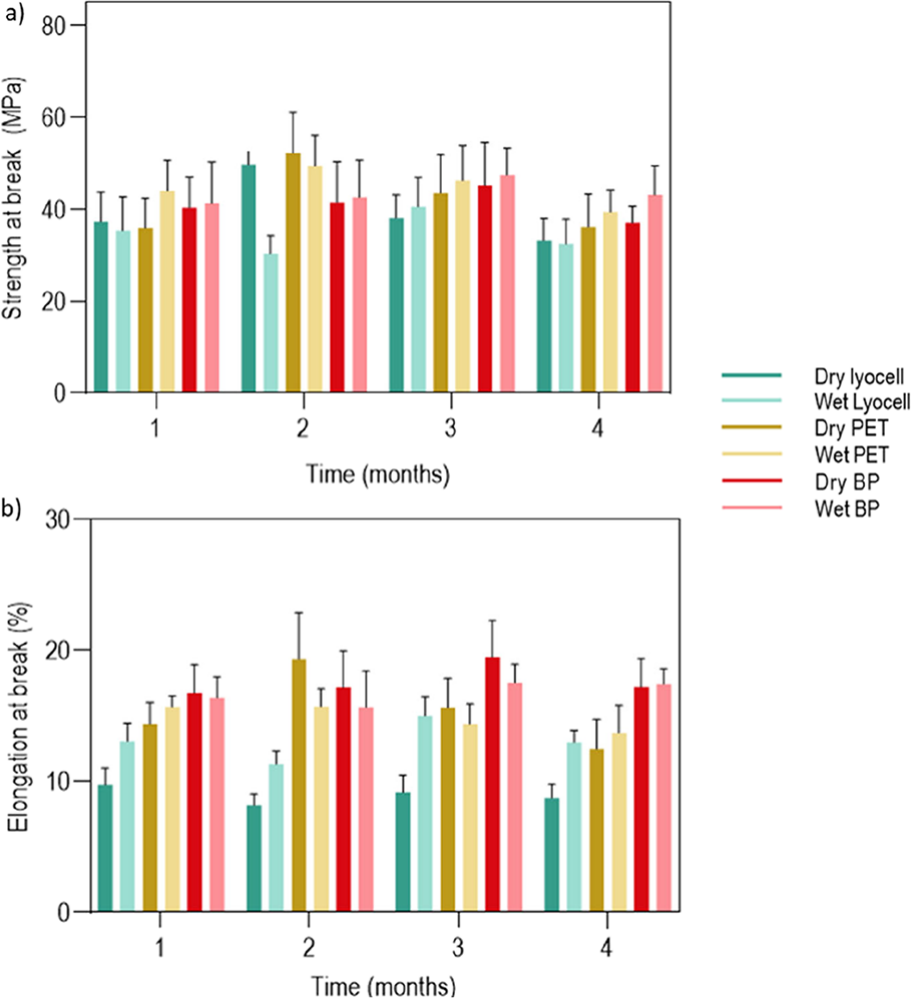
Mechanical examinations in dry and wet conditions, during 3 months of incubation: (a) variation in maximum stress at break; (b) variation in elongation (%). Statistical significance was determined by the Tukey test applying multiple comparisons between samples with the same braid, showing no significance.

**Figure 9:**
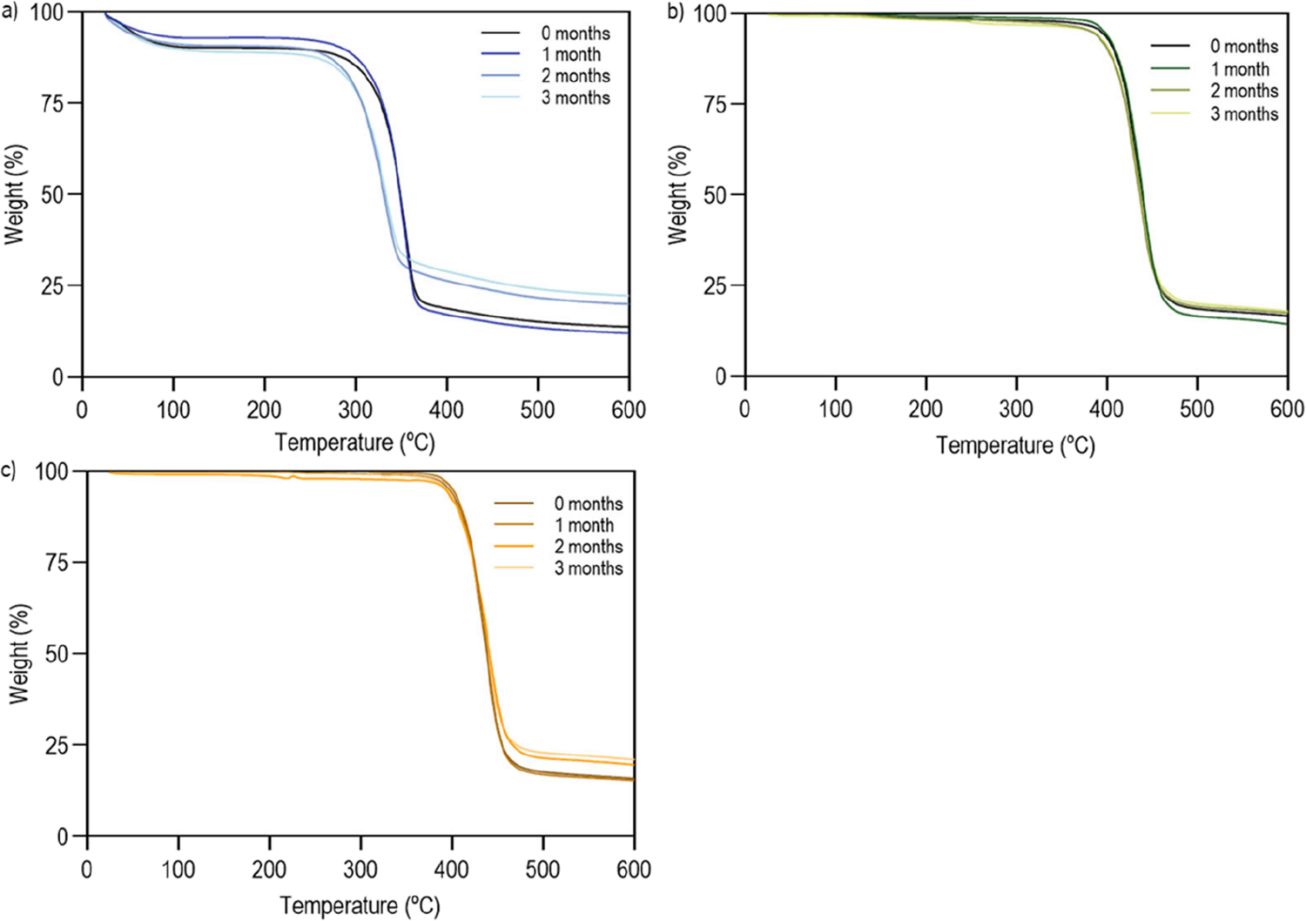
TGA thermogram of (a) lyocell, (b) PET, and (c) BP, tested over a period of 3 months, obtained under a nitrogen atmosphere at 200 mL/min.

Figure 7 reveals that mass variations did not exceed 5%, showing the stability of the polymeric braids overtime; negative values are associated with the presence of PBS salts that remained after washing.

Figure 8 shows that there are no significant differences between the same samples in different time periods, indicating that the mechanical properties are preserved throughout the study. It should be noted that, for PET and BP braids, no high degradation values were observed, either through mass reduction, reduction in tensile strength, or even reduction in thermal responses (Figure 9). These results highlight the structural stability and mechanical resistance of the materials over 3 months.^62^ Regarding lyocell, the TGA thermograms (Figure 9) demonstrate that the material underwent changes during the 3 months of incubation, thus indicating that the structural organization of the polymer altered, increasing its susceptibility to thermal degradation (peak temperature; greater mass loss occurred at temperatures below 300 °C).^62^ However, these changes were not significant enough to be felt in the mechanical properties of the material after the same period of incubation.

### 3.7 Controlled Release Profile of Cork Extract from the Braided Structures

The controlled release profile of the C2 extract was monitored by UV–vis spectroscopy, after incubating the braids, with and without extract, in 0.1 M PBS (pH 7.4) at 37 °C and 120 rpm, for 48 h. (Figure 10). Aliquots were taken after 1, 2, 4, 6, 24, and 48 h, and at each time, new PBS was added. A calibration curve was created for cork, which relates concentration to absorbance (Section S3 in Supporting Information). Based on this curve, samples were analyzed, and the results were processed by applying the difference in absorbance between the braids with extract and the braids without extract. This step eliminates possible influences of residues that could be released by the braid matrixes.^63^

**Figure 10:**
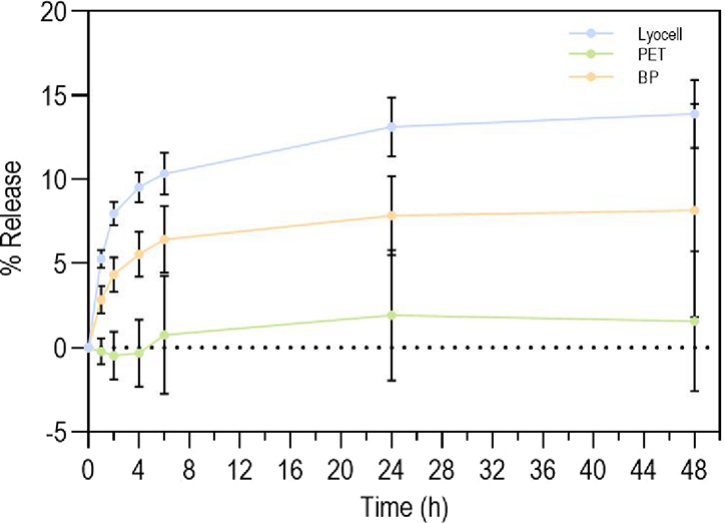
Release profile of C2 extract from lyocell, PET and BP braids, after 1, 2, 4, 6, 24, and 48 h of incubation in PBS.

The C2 extract release profile into the PBS medium presents a similar behavior among braids. After 1 h of incubation, initial release of the extract occurs in the lyocell and BP braids. Over time, up to 6 h of immersion in the medium, there is a gradual and continuous release of the extract into the medium. After 24 h, there is a stabilization in release. For the PET braid, the onset of release is observed at 6 h. This test confirms the existence of a gradual release of the extract over time, at least until 6 h of contact with PBS. Furthermore, there are differences between braids. For lyocell braid, the release profile after 48 h is 2 × higher than the release profile of BP and 13 × higher than that of PET.

These results corroborate the observations described in Sections 3.6.4 and 3.6.6, demonstrating that the differences in release profiles are likely influenced by the wettability, porosity, and structural characteristics of each matrix. While lyocell showed the highest extract release (13.90 ± 2.02%), this behavior cannot be solely attributed to its higher loading amount (0.55 ± 0.14 mg/mL; Table 6), since the data is expressed as a percentage relative to the initial concentration in each fiber. The observed differences in release profiles may be better explained by the intrinsic properties of each material. Wettability plays a crucial role, as more hydrophilic matrices tend to interact more with the surrounding medium, facilitating solvent penetration and extract diffusion. Given that lyocell is known for its high hydrophilicity, it is likely that the surrounding PBS solution exerts a stronger influence on extract release. In contrast, PET, which exhibited the lowest release (1.57 ± 4.14%), despite having an intermediate level of functionalization (0.45 ± 0.20 mg/mL; Table 6), has a lower wettability, limiting solvent absorption and thus restricting extract diffusion. Additionally, porosity affects the available surface area for solvent interaction. More porous structures provide a larger contact area, increasing the exposure of the extract to the surrounding medium and enhancing diffusion. This could explain why lyocell, which generally has a more porous structure compared to BP and PET, exhibited a significantly higher release profile. BP, despite having the lowest functionalization (0.33 ± 0.18 mg/mL; Table 6)), showed a higher release percentage than PET, which may be linked to differences in its porosity and interaction with the solvent as well. Thus, beyond just the absolute amount of extract loaded onto the fibers, the interplay between matrix properties—such as wettability and porosity—and the surrounding medium appears to be a key factor influencing the release kinetics.

### 3.8 Antioxidant Activity of Braids

The antioxidant activity of the bare and cork-functionalized braids’ was evaluated using the DPPH method.^64^ The samples were placed in contact with DPPH (ratio 1:1 cm/mL), at 37 °C, under stirring at 120 rpm, protected from light. Aliquots were collected after 1, 2, 4, 6, and 24 h of contact with DPPH and the results are shown in Figure 11.

**Figure 11:**
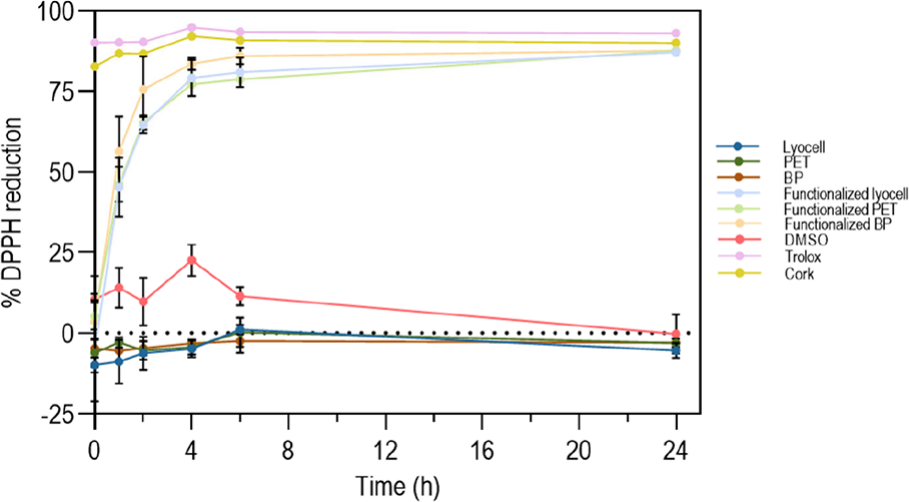
Antioxidant activity of braids with and without functionalization, C2 extract, positive (Trolox) and negative control (DMSO), after 0, 1, 2, 4, 6, 24 h in contact with DPPH. Results are presented as mean ± SD (*n* = 3).

The C2 extract followed the same trend as the Trolox control but exhibited lower DPPH reduction values. Initially, it presented a DPPH reduction of 82.79 ± 0.17%, with the highest reduction (92.09 ± 0.23%) being observed after 4 h of contact.

On the other hand, the functionalized braids had no effect in the initial moments of contact; however, after 1 h, a reduction in DPPH was observed, which continued to increase over time. After 24 h, the functionalized lyocell braid showed a DPPH reduction of 87.18 ± 1.19%, while the functionalized PET and BP braids showed a DPPH reduction of 87.61 ± 1.24 and 87.65 ± 1.16%., respectively. These findings indicate that functionalized braids can reduce DPPH by approximately 87%. The results obtained align with the release profile (Section 3.7), with an increase on DPPH reduction being dependent on the release of the C2 extracts.

The nonfunctionalized braids were tested as controls to determine whether the polymers had some antioxidant activity. Considering that the values obtained were close to zero or negative, it was concluded that the nonfunctionalized braids did not present any ability to reduce DPPH. Instead, and taking into consideration the negative values observed, the braids seem to have some ability to oxidize the DPPH instead of reducing. According to the literature, negative DPPH values may indicate interference from compounds at the wavelength, potentially caused by the release of foreign particles from the braids, or that the concentration of the compound under study is too high for the amount of DPPH to be reduced, something that does not apply in this case.^65^ In the future it will be important to carry out additional analysis to understand this phenomenon and even test different cleaning processes to verify whether the origin of such results is due to undetected residues arising from the fibers.

### 3.9 Time-Kill Kinetics

The antibacterial properties of the braids were assessed using the shake flask method, a dynamic antimicrobial evaluation in a controlled liquid environment. This quantitative test was performed against the Gram-positive bacterium *S. aureus* and the Gram-negative bacterium *P. aeruginosa*. The shake flask method is suitable for evaluating all types of textiles.^66,67^ Figure 12 shows the antibacterial activity of the samples against *S. aureus* and *P. aeruginosa* after 1, 2, 4, 6, and 24 h of culture.

**Figure 12:**
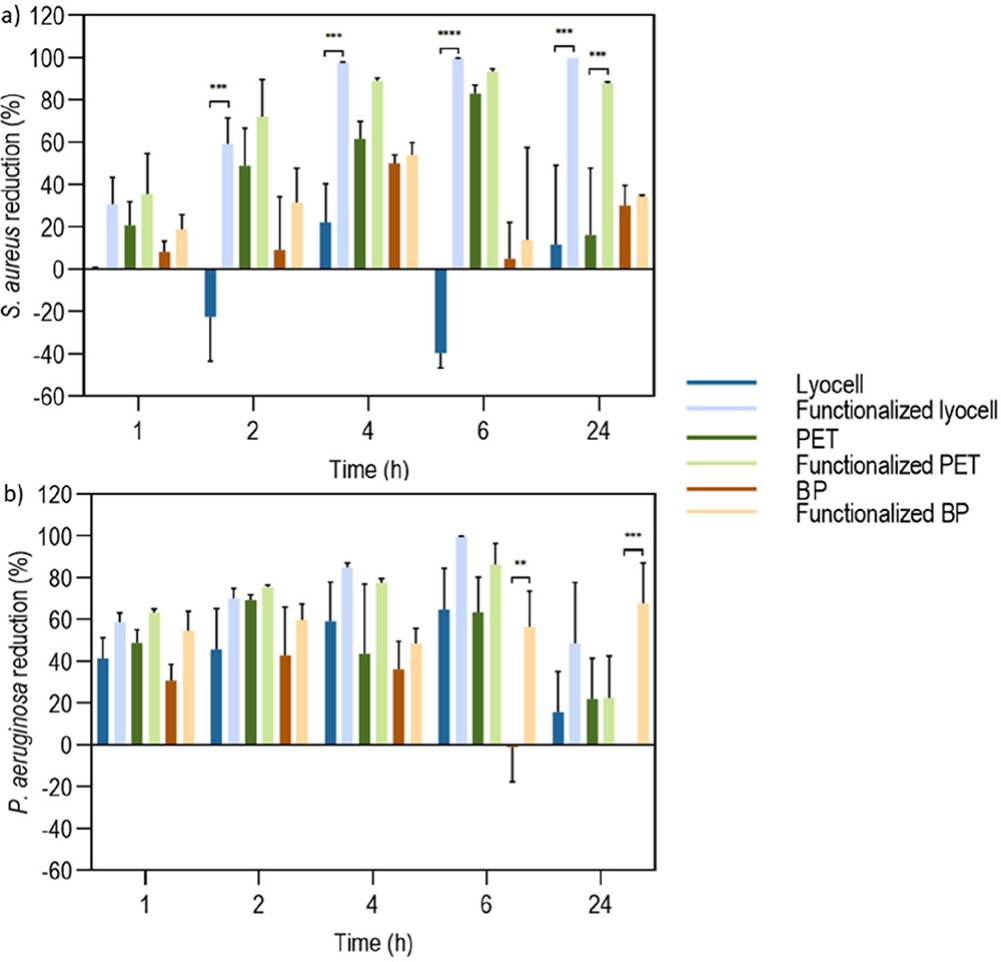
Reduction in the number of colonies of bacteria (a) *S. aureus* and (b) *P. aeruginosa*, after 1, 2, 4, 6, and 24 h of incubation at 37 °C. Data are presented as mean ± SD (*n* = 3). Statistical significance was determined by the Tukey test applying comparisons between functionalized and nonfunctionalized braids produced with the same polymer, with **p < 0.01; ***p < 0.001; ****p < 0.0001.

Initially, the bacterial reduction observed for *S. aureus*, induced by the functionalized samples was ≈30, ≈35 and ≈19%, for the lyocell, PET and BP braids, respectively (Figure 12a). As the extract is released, bacterial reduction increases, reaching values above 90% after 6 h of incubation with the samples. After 24 h, the lyocell braid functionalized with cork eliminated 99.95 ± 0.00% of *S. aureus* colonies. In contrast, functionalized BP became less effective at 6 h, attending to average value. Furthermore, it is possible to observe that, with the exception of lyocell, the PET and BP braids also showed bacterial reduction and this may be associated with the release of small residues found in the materials, despite having been washed, or the large surface area of fibrous samples that may allow bacteria to attach and, consequently, reduce their number in solution for colony counting.

For *P. aeruginosa* (Figure 12b), there was a reduction in the number of colonies in the first h of contact of the functionalized samples with the bacteria (≈60% for the lyocell braid, ≈63% for the PET braid and ≈55% for the BP). An increase of the reduction activity was observed until 6 h of incubation for all the functionalized braids, however after this period there was a significant decrease on the reduction ability for the lyocell and PET functionalized braids, while for the BP there was a gradual bacterial reduction over time reaching a 90% of reduction after 24 h. In turn, braids without functionalization, in general, demonstrated greater bacterial reduction against this bacterium. The above reasons may explain this phenomenon: greater susceptibility of the Gram-negative bacteria to potential residues present, or greater affinity of the bacteria toward the surface of the polymers under test, which could be influenced by the charge of the polymers’ surfaces. Lyocell, as a cellulose-based material, typically exhibits an anionic surface due to the presence of hydroxyl groups, which can ionize under physiological pH. PET and BP, on the other hand, are hydrophobic polyesters and do not inherently possess ionizable groups; however, their surfaces may become slightly anionic after processing or due to the adsorption of charged molecules from the environment. The anionic nature of these polymers could enhance interactions with cationic components of bacterial membranes, promoting bacterial adhesion, which might explain the observed reduction in bacterial numbers in solution for colony counting.

These results are in line with those obtained for the controlled release of the extract (Section 3.7), in which all samples from 4 h onward showed a greater release and, as such, a greater antibacterial action. It is also verified that cork displays greater effectiveness against the bacterium *S. aureus*, as expected considering the MICs and MBCs observations (Section 3.2).

### 3.10 Cytotoxicity Tests

Cytotoxicity tests via metabolic activity were carried out by direct contact between the braids and the BJ-5ta cell line for 24 and 48 h of culture (Figure 13). Taking into account the intended application, the treatment of tendon injuries, the selection of BJ-5ta was based on the predominance of tenocytes (a specific type of fibroblasts) in the tendons, responsible for the production and maintenance of the extracellular matrix components of the tendons, including collagen and proteoglycans.^68,69^

**Figure 13:**
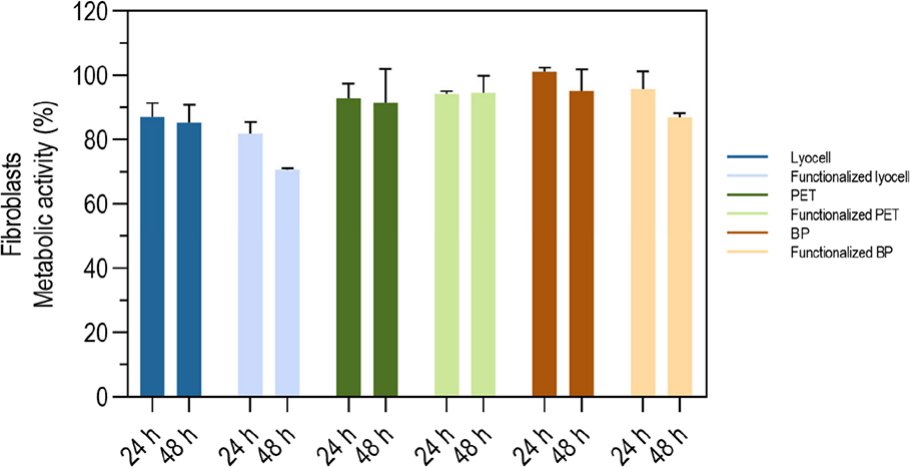
Cell viability of the human fibroblast cell line (BJ-5ta) measured via metabolic activity by the resazurin assay after 24 and 48 h of direct contact with the braids. Data are presented as mean ± SD (*n* = 2). Statistical significance was determined by the Tukey test applying multiple comparisons between braids produced with the same polymer. Results indicate the lack of significance.

The cells were exposed to culture medium following the methodology of the International Organization for Standardization (ISO) 109993-5:2009 for the biological evaluation of medical devices.^67^ According to this standard, a reduction in cell viability greater than 30% is considered an indicator of a cytotoxic effect.^70^

Comparing functionalized and nonfunctionalized braids, it can be seen that there was a reduction in metabolic activity when the extract was added (after 24 h lyocell reduced, in average, 5.15% of its metabolic activity, while after 48 h that reduction increased to 14.64%; after 24 h PET reduced, in average, 1.29% of its metabolic activity, while after 48 h that reduction increased to 2.98; after 24 h PB reduced, in average, 5.29% of its metabolic activity, while after 48 h that reduction increased to 8.19%). For example, the functionalized lyocell braid goes from 81.92 ± 3.61% at 24 h to 70.75 ± 0.40% after 48 h (a difference of 11.17%), while the functionalized BP braid decreases from 95.83 ± 5.44% at 24 h to 86.98 ± 1.39% at 48 h (a difference of 8.85%).

Interestingly, the cell viability of the functionalized PET sample increased slightly at 48 h (94.53 ± 5.45%), reaching higher values compared to the 24 h time point (92.95 ± 4.51%). A possible justification for this is the fact that lyocell and BP braids present a greater release of the C2 extract (Section 3.7).

Given that the lowest metabolic activity obtained was for functionalized lyocell (70.75 ± 0.40%), it is possible to conclude that all samples can be considered as not cytotoxic. Still, this proximity to the viability limit indicates the need for more advanced and stronger washing processes or optimization of functionalization process. In addition, the observed reduction in metabolic activity may also reflect a delayed cellular response to the material surface or to residual active compounds. Although values remained above the cytotoxicity threshold defined by ISO 10993-5, this trend could suggest transient material-induced stress or early mitochondrial adaptation. Furthermore, the use of a static culture system may have contributed to local depletion of nutrients or accumulation of extract byproducts over time. These results highlight the importance of conducting extended dynamic culture studies and possibly including other assays (e.g., live/dead staining or oxidative stress markers) in future work to better understand long-term cell-material interactions.

## 4 Conclusions and Future Perspectives

This study demonstrated the potential of biocompatible braids as a viable alternative for artificial tendon applications. Among the developed materials, the functionalized lyocell braid exhibited the most promising properties, including high mechanical strength, significant antioxidant activity, and effective antibacterial performance against *S. aureus* and *P. aeruginosa*. All tested braids were confirmed to be noncytotoxic and safe for human application; however, a reduction in metabolic activity was observed in the functionalized lyocell braid after 48 h of cell contact. Regardless, the combination of these acquired properties presents a promising strategy that addresses the critical requirements for injured hand tendons, potentially enhancing tissue regeneration while preventing infections.

Further research should focus on a more comprehensive evaluation of the bioactive and biocompatibility properties of these braided systems. Additionally, optimizing the interactions between polymers and functional extracts could further enhance their performance. Expanding antimicrobial assessments and conducting long-term cytotoxicity studies will be essential to validate their suitability for clinical applications.

## Supplementary Material



## Notes

Mechanical optimization data of lyocell, BP, and PET braids with different core structures; FTIR peak assignments for lyocell braids; UV–vis-based cork extract calibration curve and spectra; and contact angle measurements and surface free energy data for bare and cork-functionalized braids (PDF)
