# Supplementary material for: Artificial Tendons’ Responsiveness to Mechanical Stress and Biological Performance Following Cork Extract Functionalization
Source: ACS Appl Bio Mater. 2025 Aug 27;8(9):7652–71. doi: 10.1021/acsabm.5c00449 (PMC12818762; doi:10.1021/acsabm.5c00449)
Supplement: Supplementary file 1 [file mt5c00449_si_001.pdf]

Supporting Information

Artificial tendons' responsiveness to mechanical stress and biological performance following cork extract functionalization

Bruna A. S. Oliveira[1,2], Marta O. Teixeira[1], Sónia P. Gonçalves[1], Artur Ribeiro[2,3], Carla Silva[2,3], Helena P. Felgueiras[1,*]

[1]Centre for Textile Science and Technology (2C2T), University of Minho, Campus of Azurém, 4800-058 Guimarães, Portugal; pg49861@alunos.uminho.pt (B.A.S.O.); martaoliveirateixeira@2c2t.uminho.pt (M.O.T); sonia.pires@2c2t.uminho.pt (S.P.G.); helena.felgueiras@2c2t.uminho.pt (H.P.F.)

[2]Centre of Biological Engineering (CEB), University of Minho, Campus of Gualtar, 4710-057 Braga, Portugal; arturibeiro@ceb.uminho.pt (A.R.); carla.silva@ceb.uminho.pt (C.S.)

[3]LABBELS – Associate Laboratory, 4710-057 Braga, Guimarães, Portugal

*Correspondence: helena.felgueiras@2c2t.uminho.pt; Tel.: +351-253-510-283; Fax: +351-253-510-293

Section S1 - Optimization of braids' production

Braids' production was optimized in light of their mechanical performance. Here, three materials, lyocell, BP and PET, were processed in the form of braids without a core and with a core (4, 8, 16, 32 loose yarns or in the form of a braid of 16 yarns). Tensile testing was conducted, and the results were reported in Table S1 for lyocell, Table S2 for BP and Table S3 for PET.

Table S1. Tensile testing of lyocell braids form of yarns with 14 v/''torsion, where v represents the winding speed of the braids. Results were presented as mean ± SD (n=15)

Braided	Maximum strength (N)	Elongation at break (%)	Strength at break (MPa)
Braided exterior with v=3.0	267.87 ± 5.10	25.38 ± 3.35	107.39 ± 21.03
Braided exterior with v=3.0 and core with 4 loose threads	212.67 ± 38.97	11.84 ± 2.26	56.55 ± 15.70
Braided exterior with v=3.0 and core with 8 loose threads	242.53 ± 25.00	10.85 ± 1.50	63.14 ±16.70
Braided exterior with v=3.0 and core with 16 loose threads	297.33 ± 24.84	11.86 ± 2.80	68.43 ± 13.88
Braided exterior with v=3.0 and core with 32 loose threads	337. 73 ± 50.38	8.72 ± 2.05	53.15 ± 12.62
Braided exterior with v=3.0 and core with braided v=3.0	303.60 ± 45.65	10.25 ± 2.36	73.18 ± 17.64

Table S2. Tensile testing of BP braids, where v represents the winding speed of the braids. Results were presented as mean ± SD (n=15)

Braided	Maximum strength (N)	Elongation at break (%)	Strength at break (MPa)
Braided exterior with v=3.0	366.93 ± 54.85	28.31 ± 4.96	56.76 ± 13.44
Braided exterior with v=3.0 and core with 4 loose threads	293.27 ± 77.45	18.72 ± 6.58	51.57 ± 15.91
Braided exterior with v=3.0 and core with 8 loose threads	320.53 ± 56.39	18.67 ± 3.58	44.66 ± 11.29
Braided exterior with v=3.0 and core with 16 loose threads	408.73 ± 22.68	19.14 ± 1.13	43.26 ± 6.18
Braided exterior with v=3.0 and core with 32 loose threads	551.73 ± 102.47	19.01 ± 3.97	38.91 ± 8.22
Braided exterior with v=3.0 and core with braided v=3.0	445.60 ± 42.49	19.93 ± 3.27	35.01 ± 5.77

Table S3. Tensile testing of PET braids, where v represents the winding speed of the braids. Results were presented as mean ± SD (n=15)

Braided	Maximum strength (N)	Elongation at break (%)	Strength at break (MPa)
Braided exterior with v=3.0	137.29 ± 45.51	24.84 ± 14.07	75.82 ± 29.58
Braided exterior with v=3.0 and core with 4 loose threads	120.37 ± 31.75	13.00 ± 6.49	73.19 ± 26.76
Braided exterior with v=3.0 and core with 8 loose threads	124.93 ± 26.06	10.63 ± 3.56	60.47 ± 13.61
Braided exterior with v=3.0 and core with 16 loose threads	157.93 ± 48.89	10.88 ± 6.17	55.24 ± 17.80
Braided exterior with v=3.0 and core with 32 loose threads	214.53 ± 65.87	10.66 ± 5.04	44.12 ± 12.02
Braided exterior with v=3.0 and core with braided v=3.0	218.20 ± 77.13	19.04 ± 10.78	69.78 ± 32.46

Section S2 - Complementary data to FTIR analysis

Table S4 presents the wavelengths of the lyocell braid, the corresponding chemical bonds, and their associated components. Using these data alongside the analysis provided in Figure 2, it becomes easier to interpret the ATR-FTIR spectrum and confirm that the braid is composed of lyocell.

Table S4. List of characteristic peaks identified in the ATR-FTIR spectrum of the lyocell braids and their respective functional groups [1]. N-methylmorpholine-N-oxide (NMMO) is the solvent used in the lyocell production process.

Wavenumber (cm^{-1})	Functional Groups Assigned	Component
2890	CH_2 asymmetric stretching	Cellulose, NMMO
1418	CH_2 symmetric bending	Cellulose, NMMO
1363,1263	CH bending	Cellulose, NMMO
1313	CH_2 waggiing	Cellulose, NMMO
1198	OH in plane bending	Cellulose, NMMO
1155, 1022	stretching C-O	Cellulose, NMMO

Section S3 - Cork calibration curve

A cork extract calibration curve was prepared using varying concentrations of cork extract, namely 2.0; 5.0; 10.0; 11.0; 16.0; 24.0; 32.0 µg/mL. Samples were analyzed by UV-Vis spectroscopy, in a wavelength range between 190 nm and 500 nm, in order to determine the cork absorption peak, as illustrated in Figure S1. The sharpest peak was observed at 197 nm, which data was used for constructing the calibration curve shown in Figure S2. However, according to the literature, cork's absorption peak is located at 280 nm. In those cases, the spectral sweep was between 200 and 600 nm, not covering lower wavelengths, such as 197 nm [2–4].

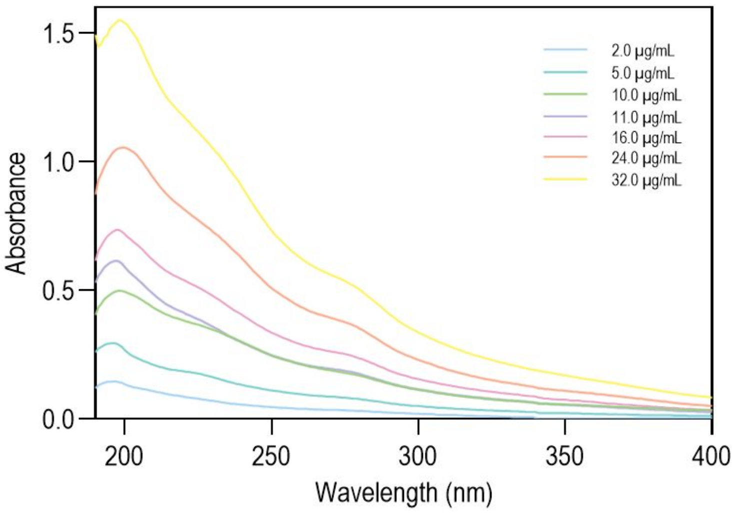

Figure S1. UV-Vis spectroscopy profile of cork extract for different concentrations.

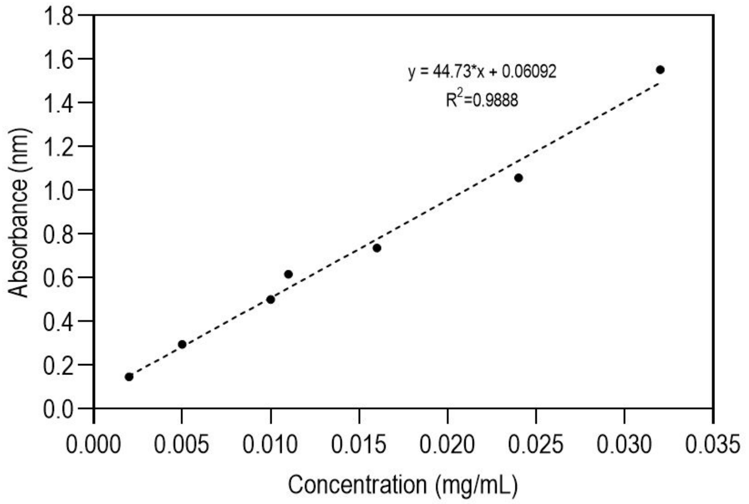

Figure S2. Cork extract calibration curve.

Section S4 - Determination of surface free energy

In order to calculate the surface free energy, contact angles were measured with three liquids on cork-functionalized and bare braiding system (Table S4).

Table S4. Contact angles (°) obtained with water, ethylene glycol and diiodomethane on bare and cork extract-functionalized braids, used for calculating the surfaces free energy (n=5)

Sample	Water (°)	Ethylene glycol (°)	Diiodomethane (°)
Lyocell	0	0	0
Functionalized lyocell	0	0	0
PET	137.91 ± 8.54	41.74 ± 3.63	0
Functionalized PET	114.60 ± 13.69	41.74 ± 10.42	0
BP	152.28 ± 12.67	78.78 ± 8.29	0
Functionalized BP	147.07 ± 10.06	65.76 ± 15.04	0
